# The antibacterial activity of peptide dendrimers and polymyxin B increases sharply above pH 7.4†

**DOI:** 10.1039/d1cc01838h

**Published:** 2021-05-05

**Authors:** Xingguang Cai, Sacha Javor, Bee Ha Gan, Thilo Köhler, Jean-Louis Reymond

**Affiliations:** Department of Chemistry, Biochemistry and Pharmaceutical Sciences, University of Bern Bern Switzerland jean-louis.reymond@dcb.unibe.ch; Department of Microbiology and Molecular Medicine, University of Geneva Geneva Switzerland

## Abstract

pH-activity profiling reveals that antimicrobial peptide dendrimers (AMPDs) kill *Klebsiella pneumoniae* and Methicillin-resistant *Staphylococcus aureus* (MRSA) at pH = 8.0, against which they are inactive at pH = 7.4, due to stronger electrostatic binding to bacterial cells at higher pH. A similar effect occurs with polymyxin B and might be general for polycationic antimicrobials.

## Abstract 2

A previously unknown pH-effect on the antimicrobial activity of peptide dendrimers and polymyxin B against *Klebsiella pneumoniae* and MRSA is reported.

## Graphical abstract

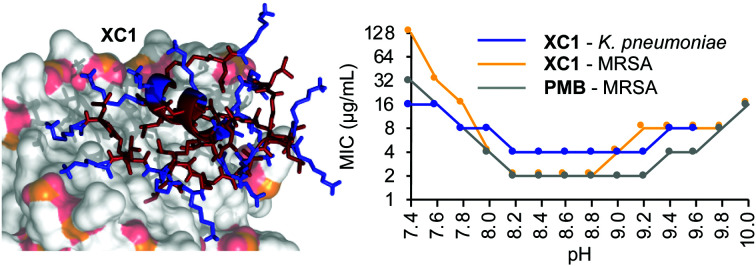

Dendrimers are tree-like macromolecules useful for a broad range of applications.^1–3^ In our efforts to develop antimicrobial dendrimers^4^ against ESKAPE pathogens,^5^ we recently showed that peptide dendrimers^6–8^ consisting of lysine and leucine such as **G3KL** and **T7** kill Gram-negative bacteria including multidrug resistant strains (Fig. 1).^9–11^ Similar to many antimicrobial peptides (AMPs),^12–15^ polymers,^16^ peptidomimetics^17^ and foldamers,^18^ our peptide dendrimers act by a membrane disruptive mechanism,^19^ which in our case involves α-helical folding of the amphiphilic dendrimer core in contact with the bacterial membrane.^20^

**Fig. 1:**
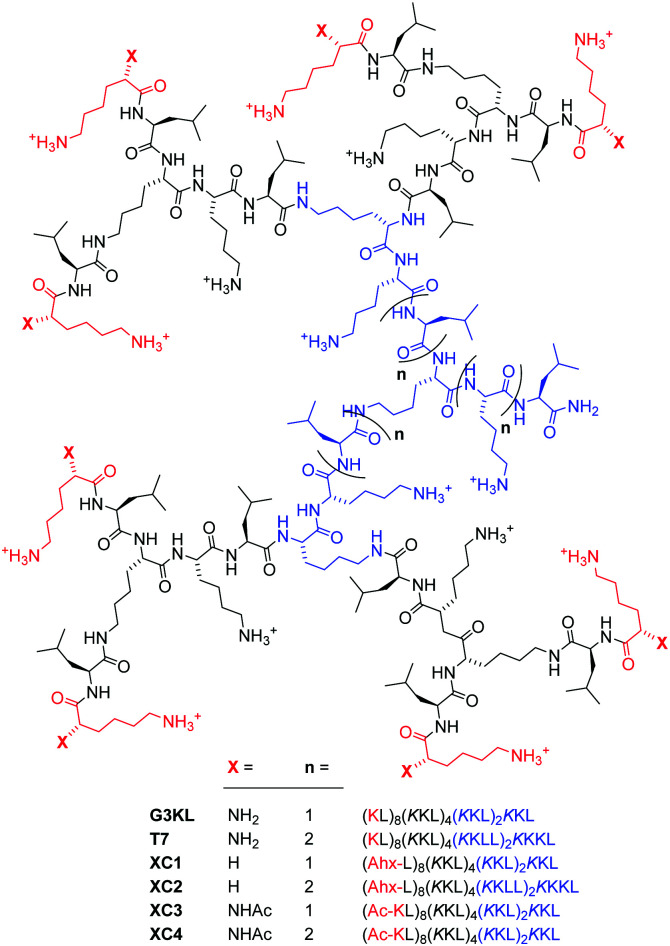
Structure of peptide dendrimers investigated in this study. Ahx = aminohexanoic acid.

**G3KL** and **T7** possess eight N-termini with a depressed p*K*_a_ of ∼6.5 due to multivalency, implying that, as for related transfection dendrimers,^21,22^ the number of positive charges strongly increases at acidic pH.^23^ We therefore set out to test if their activity might be pH dependent, an effect observed with many AMPs,^24^ and which is important since sites of bacterial infections may be acidic (biofilms, skin surface), or basic (chronic wounds).^25,26^ For our AMPDs, activity might increase at low pH as reported for clavanins, which are AMPs containing histidine side-chains (p*K*_a_ ∼6),^27^ or decrease due to unfolding of membrane-disruptive conformations and increased proteolytic degradation as reported for AMPs such as LL-37 or lactoferrin.^28^

Anticipating a major role of N-termini in the possible pH-dependent activity of our AMPDs, we prepared analogs **XC1–XC4** in which these N-termini have been either removed or acetylated (Fig. 1). As expected, their titration curves lacked the plateau observed with **G3KL** and **T7** around pH 6.5 (Fig. S1a and S1b, ESI†). Circular dichroism (CD) spectra of **XC1–XC4** were similar at pH 7.4 and pH 8.0 and comparable to those of **G3KL** and **T7**, indicating a transition from a random coil in aqueous buffer to an α-helical trace upon addition of 5 mM dodecylphosphocholine (DPC) or 10 mM sodium dodecyl sulfate (SDS) mimicking membrane environments. In contrast to the CD traces of **G3KL** and **T7**, however, the CD traces of **XC1–XC4** remained almost unchanged upon acidification to pH 5.0 (Fig. 2a, Fig. S2 and S3, ESI†).

**Fig. 2:**
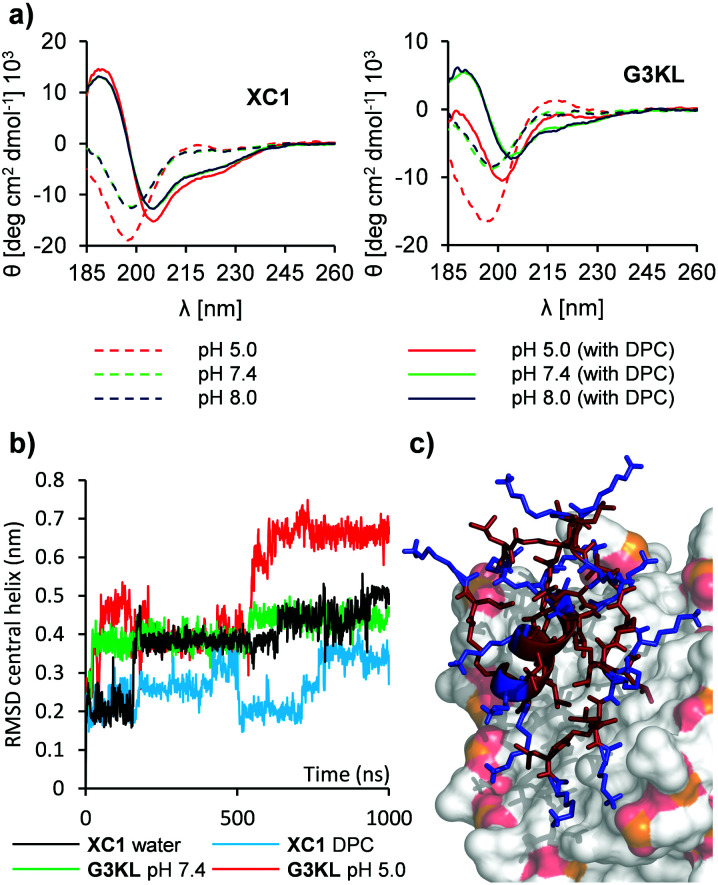
CD spectroscopy and MD simulations with AMPDs at different pH values. (a) CD spectra of **XC1** and **G3KL** (0.100 mg mL^−1^ of dendrimer) in aq. buffer (10 mM acetate pH 5.0, 10 mM phosphate pH 7.4 or pH 8.0) with or without 5 mM DPC. See methods for details. (b) RMSD of the central α-helix over the course of the MD simulations in water for **XC1**, **G3KL** with neutral N-termini (pH 7.4) or with protonated N-termini (pH 5.0). (c) MD simulation of **XC1** with a DPC micelle after 150 ns. Lys and Ahx in blue, Leu, branching Lys in red, DPC in surface representation with C in grey, O in red, N in blue and P in orange.

The CD data was supported by molecular dynamics (MD) simulations using GROMACS^29^ with **XC1** and **G3KL**. Starting from a fully α-helical conformation in water, **XC1** and **G3KL** with neutral N-termini unfolded in water at a similar rate. On the other hand, **G3KL** with protonated N-termini unfolded significantly faster, suggesting that this protonation triggered destabilization of the central α-helix as observed at low pH with **G3KL** but not with **XC1** (Fig. 2b). Furthermore, the core amphiphilic α-helix of **XC1** was preserved in MD simulations run in the presence of a DPC micelle, and formed a large hydrophobic patch also involving leucine residues from other branches of the dendrimer. In this model, leucine residues were directly sitting on top of the lipid tails of DPC while lysine side-chain ammonium groups interacted either with phosphate groups or with the solvent, providing a pH independent model for dendrimer membrane interactions (Fig. 2c).

To test the possible pH dependent activity of the various AMPDs, we determined minimum inhibitory concentrations (MIC) in Müller-Hinton (MH) culture medium adjusted to pH 5.0, pH 7.4 and pH 8.0 against four Gram-negative and one Gram-positive bacteria (Table 1). As control, we detected known pH dependencies such as the increased activity of azithromycin and ciprofloxacin at basic pH, an effect attributed to better membrane permeation of their neutral form at higher pH,^30,31^ and also reported with high bicarbonate with azithromycin (Fig. S4 and Table S2, ESI†).^32^ In this assay, the activity of **G3KL** and **T7** against *Escherichia coli*, *Acinetobacter baumannii* and *Pseudomonas aeruginosa* at pH 7.4 (MIC = 4–8 μg mL^−1^) increased at pH 8.0 (MIC = 1–4 μg mL^−1^) but decreased upon acidification to pH 5.0 (MIC = 16–32 μg mL^−1^). The effect was even more pronounced with *Klebsiella pneumoniae* and methicillin-resistant *Staphylococcus aureus* COL (MRSA), against which the dendrimers switched from inactive at pH 5.0 and pH 7.4 to MIC = 2–8 μg mL^−1^ at pH 8.0. These data suggested that **G3KL** and **T7** were more active with their N-termini as free base and that disabling their protonation might enable pH-independent antibacterial activity.

**Table 1:** pH dependent antimicrobial activities (MIC at pH 5.0/pH 7.4/pH 8.0) of peptide dendrimers and polymyxin B.^a^

Cpd	*E. coli* W3110	*A. baumannii* ATCC 19606	*P. aeruginosa* PAO1	*K. pneumoniae* NCTC 418	MRSA COL	MHC
**G3KL**	32/8/1–2	8/8/1	16/4/1	>64/>64/4	>64/>64/2	>2000
**T7**	16/4/2	16/8/2–4	16/8/2–4	>64/32/8	>64/>64/4	>2000
**XC1**	2/2/1–2	1/2/2	8/4/2	16/16/2–4	>64/>64/2	>2000
**XC2**	4/8/4	4/4/4	16/8/4	32/32/8–16	>64/>64/4	31.25
**XC3**	2/4/1	4/2/2	>64/4/2	>64/>64/4	>64/>64/8	>2000
**XC4**	2/4/2	2/2/2	32/8/2–4	>64/>64/8	>64/>64/4	>2000
**PMB**	0.02/0.25/0.13	1/0.25/0.25	0.03/0.5/0.5	8/0.25/0.25	>64/>64/4	>2000
**G3KL-Fluo**	8/2/16	4/4/16	8/4/16	>64/>64/8	>64/>64/64	N/A

a MIC = minimal inhibitory concentration in μg mL^−1^, measured in Müller–Hinton (MH) medium at pH 5.0/7.4/8.0 on *E. coli, A. baumannii, P. aeruginosa, K. pneumoniae* and MRSA (methicillin-resistant *Staphylococcus aureus*) after incubation for 16–20 h at 37 °C. Minimum hemolytic concentration (MHC) measured on human red blood cells in phosphate buffered saline pH 7.4 at room temperature for 4 h. Each result represents two independent experiments performed in duplicate.

Indeed, the four modified dendrimers **XC1–XC4** showed an almost pH-independent activity against *E. coli* and *A. baumannii*. Furthermore, removing N-termini did not affect hemolysis except for **XC2** (Table 1, Fig. S5 and Table S3, ESI†). On the other hand, **XC1–XC4** behaved similarly to **G3KL** and **T7** against *P. aeruginosa*, *K. pneumoniae* and MRSA and only showed strong activity at pH 8.0. This observation indicated that factors other than the ionization state of N-termini influenced the activity of our AMPDs. In fact, we found that the cyclic peptide polymyxin B (**PMB**), which is inactive against most MRSA strains unless structurally modified,^33–35^ also switched from inactive at pH 7.4 to active at pH 8.0 against this bacterium although its five primary ammonium side chains do not change protonation state around neutral pH (Fig. S1c, ESI†). Activity increase with pH without change in protonation state has also been reported for three specific short linear AMPs.^24^

Among all dendrimers, **XC1** consistently showed the strongest activity across all strains tested and retained the best activity at pH 5.0. Activity was verified by time-kill experiments at 4 × MIC at the various pH values (Fig. 3a and Fig. S6a, ESI†). Time-kill experiments also confirmed the strong activity increase of **XC1** at basic pH against *K. pneumoniae* and MRSA, an effect also observed with **G3KL** and **PMB** (Fig. 3b and Fig. S6b, ESI†). Transmission electron microscopy (TEM) images upon exposure of *K. pneumoniae* cells at pH 8.0 showed membrane disruption with all three compounds, however the effect was less visible with MRSA, probably because the thick peptidoglycan layer better preserves the cellular shapes in this Gram-positive bacterium (Fig. 3c and Fig. S7–S14, ESI†). A pH-activity profile with **XC1** and **PMB** showed that the strongest activity occurred in the pH interval 8.2–9.2 (Fig. 3d).

**Fig. 3:**
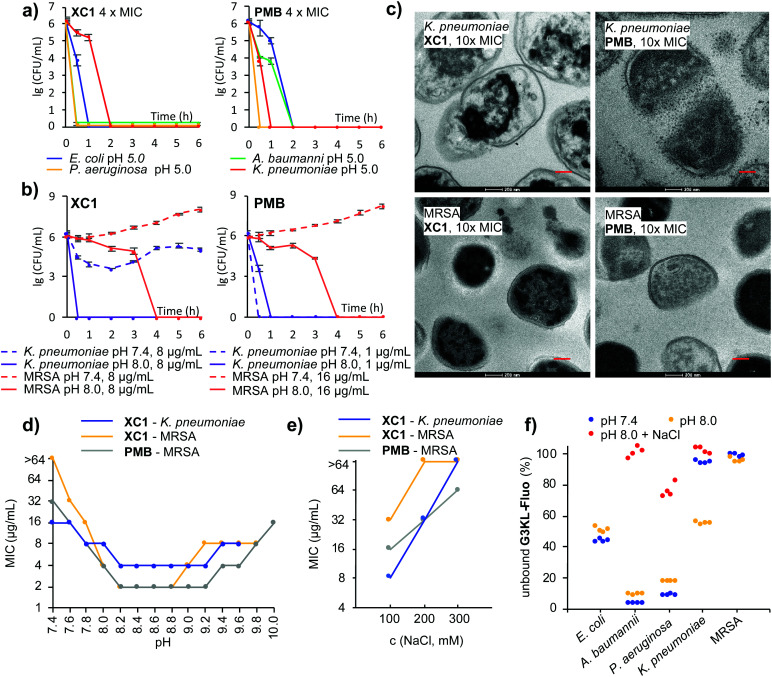
(a) Bacteria killing assay at pH 5.0 against *E. coli, A. baumannii, P. aeruginosa* PAO1 and *K. pneumoniae* at a concentration of 4 × MIC. (b) Bacteria killing assay at pH 7.4 and pH 8.0 against *K. pneumoniae* and MRSA. (c) TEM images of *K. pneumoniae*, 2 h after treatment of **XC1**, **G3KL** and **PMB** in MH medium at pH 8.0. Scale bar is 200 nm. (d) MIC of **XC1** against *K. pneumoniae* and MRSA at different pH, MIC of **PMB** against MRSA at different pH. Growth of bacteria was not observed above the shown pH. (e) MIC of **XC1** against *K. pneumoniae* and MRSA at pH 8.0 in presence of NaCl, MIC of **PMB** against MRSA at pH 8.0 in presence of NaCl. (f) Quantification of unbound **G3KL-Fluo** (40 μg mL^−1^) in the presence of 10^9^ CFU mL^−1^ (OD_600_ = 1) of *E. coli, A. baumannii, P. aeruginosa* PAO1, *K. pneumoniae* and MRSA for 2 hours. 300 mM NaCl was added when specified. In figures d and e, each result represents two independent experiments performed in duplicate.

Considering that resistance to AMPDs, although difficult to trigger, is similar to resistance to **PMB** and mediated by changes reducing the net negative charge of the bacterial outer membrane,^36,37^ the increased activity with pH might result from an increase in negative charge density at the bacterial surface, *e.g.* by deprotonation of phosphate groups, leading to stronger binding to polycationic compounds.^24^ Indeed, the activity of **XC1** against *K. pneumoniae* and MRSA and of **PMB** against MRSA at pH 8.0 decreased with increasing salt concentration, an effect often observed with polycationic AMPs and consistent with an electrostatic interaction (Fig. 3e, Table 1 and Table S1, ESI†).^24^ To show that the pH and ionic strength dependent activity changes reflected modulation of binding to the bacteria, we used the fluorescein-labeled dendrimer **G3KL-Fluo**^23^ and assessed its binding to bacteria by quantifying unbound **G3KL-Fluo** by residual fluorescence of the cell culture medium after centrifugation of bacterial cells (Fig. 3f and Fig. S15, ESI†). In the case of *E. coli, A. baumannii* and *P. aeruginosa* against which **G3KL-Fluo** was slightly less active at pH 8.0 than at pH 7.4 (Table 1), binding was comparable at both pH values but was strongly reduced with added NaCl. For *K. pneumoniae* against which **G3KL-Fluo** was inactive at pH 7.4, active (MIC = 8 μg mL^−1^) at pH 8.0, but again inactive with high salt, binding to the bacteria correspondingly increased between pH 7.4 and pH 8.0 but was abolished by addition of 300 mM NaCl. On the other hand, there was no significant binding of **G3KL-Fluo** to MRSA cells at both pH values, in line with the fact that **G3KL-Fluo** remained inactive against MRSA at both pH values.

In summary, although the pH-dependence of activity of AMPs and small molecule antibiotics was well documented,^24,27,28,30,31^ our study revealed a previously unknown activity increase between pH 7.4 and pH 8.0 against *K. pneumoniae* and MRSA with AMPDs and **PMB**. pH-profiling of polycationic AMPs and analogs such as dendrimers,^4^ polymers,^16^ peptidomimetics^17^ and foldamers^18^ might reveal related effects and increase the application potential of such compounds, in particular when considering topical treatment where local buffering can be considered.

This work was supported by the Swiss National Science Foundation (grant no. 200020_178998) and the European Research Council (grant no. 885076). The authors thank T. N. Siriwardena, D. Erzina and M. Heitz for helpful discussion.

## Conflicts of interest

There are no conflicts to declare.

## Supplementary Material

CC-057-D1CC01838H-s001
