# Supplementary material for: The antibacterial activity of peptide dendrimers and polymyxin B increases sharply above pH 7.4
Source: Chem Commun (Camb). 2021 May 5;57(46):5654–7. doi: 10.1039/d1cc01838h (PMC8186529; doi:10.1039/d1cc01838h)
Supplement: CC-057-D1CC01838H-s001 [file CC-057-D1CC01838H-s001.pdf]

Electronic Supplementary Material (ESI) for ChemComm.
This journal is © The Royal Society of Chemistry 2021

Electronic Supplementary Information for:

The Antibacterial Activity of Peptide Dendrimers and Polymyxin B Increases Sharply Above pH 7.4

Xingguang Cai, [a] Sacha Javor, [a] Bee-Ha Gan, [a] Thilo Köhler [b, c] and Jean-Louis Reymond [a] *

[a] *Department of Chemistry, Biochemistry and Pharmaceutical Sciences, University of Bern, Freiestrasse 3, 3012 Bern, Switzerland;* [b] *Department of Microbiology and Molecular Medicine, University of Geneva;* [c] *Service of Infectious Diseases, University Hospital of Geneva, Geneva, Switzerland*

E-Mail: jean-louis.reymond@dcb.unibe.ch

Table of Contents

1. Solid phase synthesis of peptide dendrimers 3

2. Acid-base titration 17

3. Circular dichroism (CD) spectroscopic measurements 18

4. Molecular Dynamics (MD) 21

    4.1 Parameters for the non-natural residue aminohexanoic acid (Ahx) 22

    4.2 MD in the presence of a DPC micelle 23

5. MIC determination with different pH values and ionic strength 26

6. Relative antibiotics 28

7. Hemolysis assay 30

8. Time kill kinetics assay 32

9. Transmission electron microscopy (TEM) 34

10. Quantification of bacterial binding of G3KL-Fluo 43

1. Solid phase synthesis of peptide dendrimers

Dimethylformamide (DMF) was purchased from Thommen-Furler AG, Buren, Switzerland. Dichloromethane (DCM), methanol and *tert*-butylmethylether (TBME) were purchased from Dr. Grogg Chemmie AG, Stettlen-Deisswil, Switzerland. Piperidine was purchased from Acros Organics, Geel, Belgium. *N,N′*-Diisopropylcarbodiimid (DIC) and Boc-*6*-Ahx-OH was purchased from Iris biotech GMBH Markredwitz, Germany. Trifluoroacetic acid (TFA) and triisopropylsilane (TIS) was purchased from fluorochem Ltd., Hadfield, U. K. *2,4,6*-trinitrobenzenesulfonic acid (TNBS) was purchased from Sigma-Aldrich cheimie GmbH, Steinheim, Germany. TentaGel S RAM resin was purchased from Rapp Polymere GmbH, Tübingen, Germany.

Peptide synthesis was carried out manually with TentaGel S RAM resin (0.22 mmol/g). Firstly, resin was swelled in DCM and the Fmoc-protecting groups of the resin were removed with a solution of 20% piperidine in DMF (2 × 10 min). For further couplings, the resin was acylated with one of the protected amino acids (5 eq./amine), OxymaPure (6 eq./amine) and DIC (6 eq./amine) in DMF. Fmoc-protected amino acids, derivatives or diamino acids were coupled for two times 1 h (G0), two times 1 h (G1), three times 2 h (G2) and three times 2 h + one time overnight (G3). The completion of the reaction was checked using TNBS. The coupling was repeated after a positive test. After each coupling, the resin was deprotected with 20% piperidine in DMF (2 × 10 min).

Final deprotection was done in (20% piperidine in DMF, 2 × 10 min) by manually after the synthesis. The resin was washed twice with MeOH and dried under vacuum before the cleavage was carried out using TFA/TIS/H_2O (94:5:1 v/v/v) for 4.5 h. After filtration, the peptide was precipitated with 50 mL ice cold TBME, centrifuged at 4400

rpm for 10 min, and washed twice with TBME. For purification of the crude peptide, it was dissolved in A (100% mQ-H_2O, 0.05% TFA), subjected to preparative RP-HPLC and obtained as TFA salt after lyophilization. B was 10% mQ-water, 90% acetonitrile, 0.05% TFA.

XC1 ((Ahx-L)$_8$(*K*KL)$_4$(*K*KL)$_2$*K*KL) was obtained from TentaGel S RAM resin (363.6 mg, 0.08 mmol, 0.22 mmol·g^{-1}), the dendrimer was obtained as a white foamy solid after preparative RP-HPLC purification (158.3 mg, 32.3%). Analytical RP-HPLC: t_R = 1.67 min (100% A to 100% B in 3.5 min, λ= 214 nm). MS (ESI+): $C_{222}H_{424}N_{52}O_{37}$ calc./obs. 4411.29/4411.35 [M]$^+$.

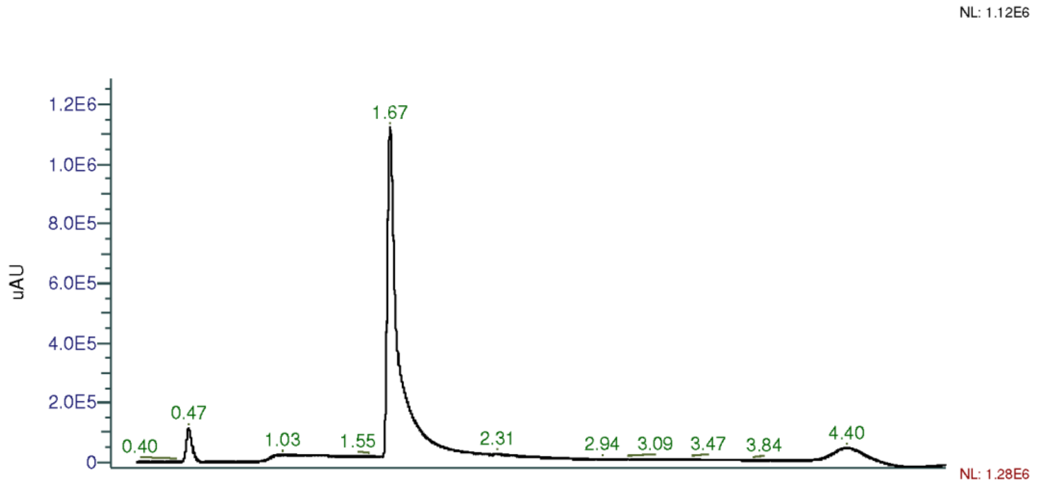

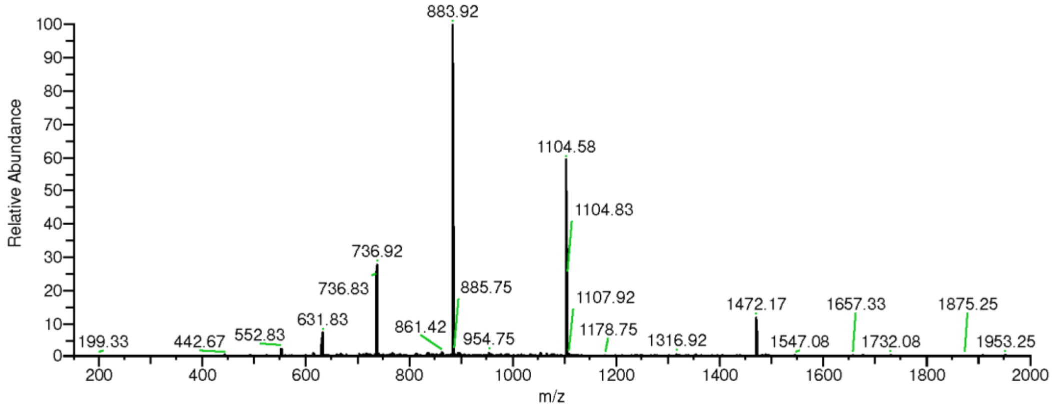

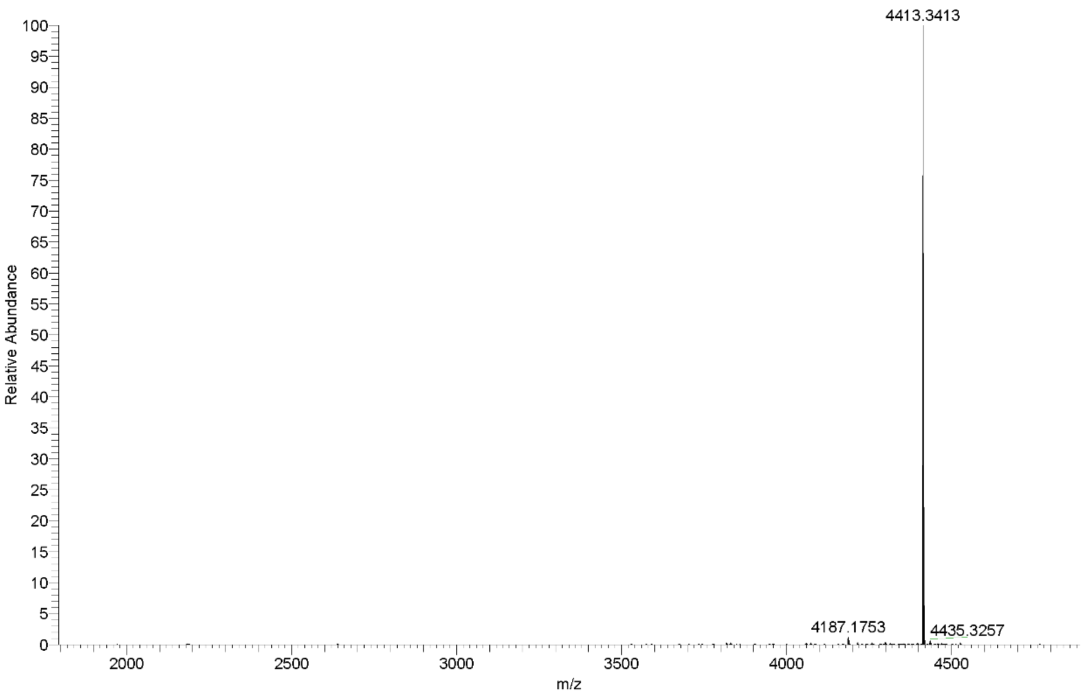
4413.3413
4187.1753
4435.3257
Relative Abundance
m/z

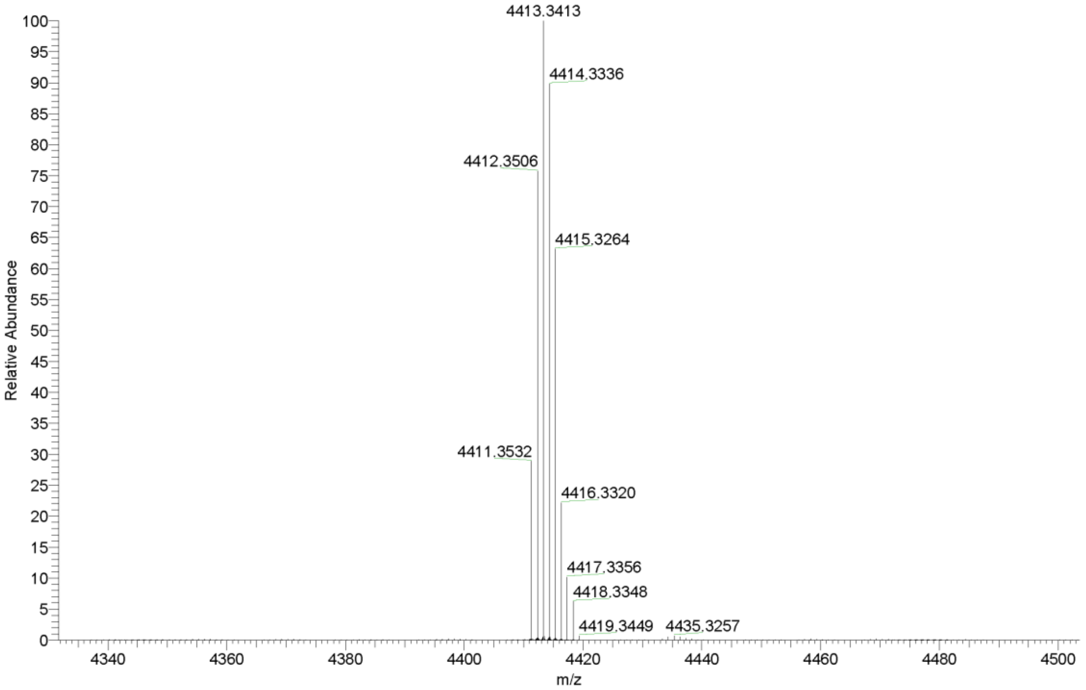
4413.3413
4414.3336
4412.3506
4415.3264
4411.3532
4416.3320
4417.3356
4418.3348
4419.3449
4435.3257
Relative Abundance
m/z

XC2 ((Ahx-L)$_8$(*K*KL)$_4$(*K*KLL)$_2$*K*KKL) was obtained from TentaGel S RAM resin (363.6 mg, 0.08 mmol, 0.22 mmol·g^{-1}), the dendrimer was obtained as a white foamy solid after preparative RP-HPLC purification (106.5 mg, 20.2%). Analytical RP-HPLC: t_R = 1.78 min (100% A to 100% B in 3.5 min, λ= 214 nm). MS (ESI+): $C_{240}H_{458}N_{56}O_{40}$ calc./obs. 4765.55/4765.57 [M]$^+$.

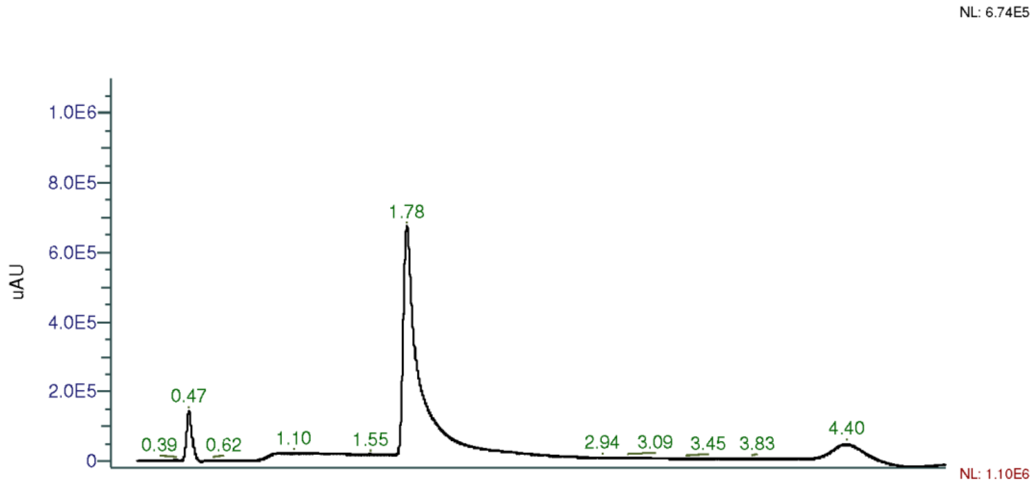

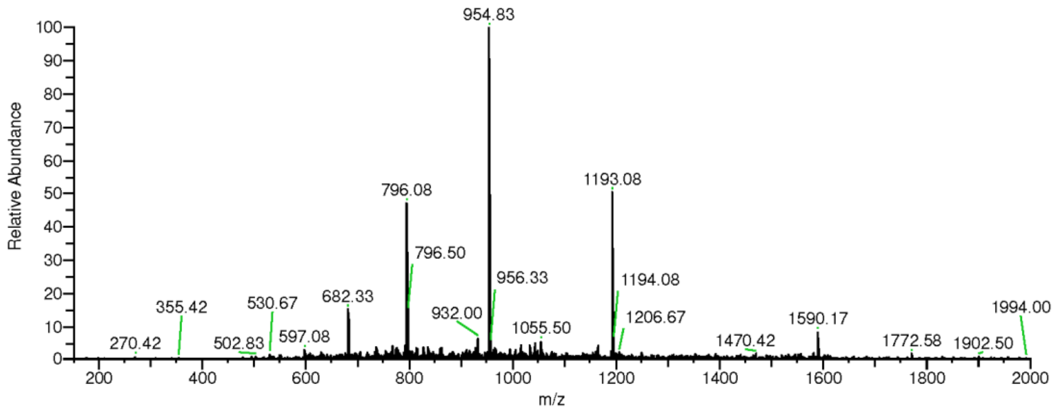

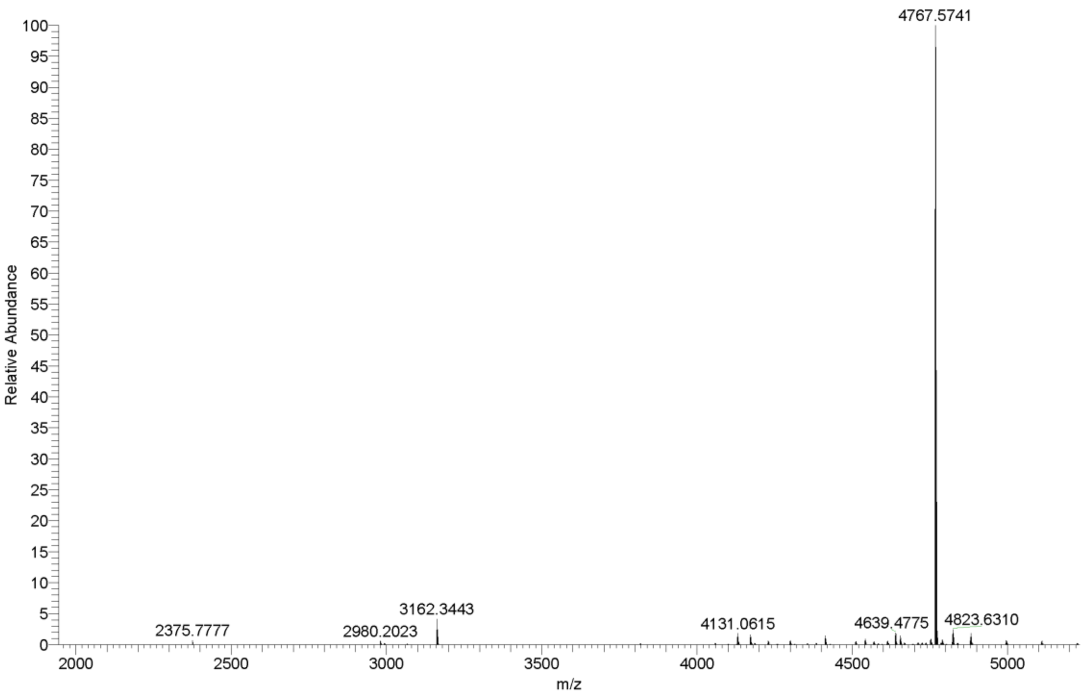
4767.5741
Relative Abundance
3162.3443
2375.7777
2980.2023
4131.0615
4639.4775
4823.6310
m/z

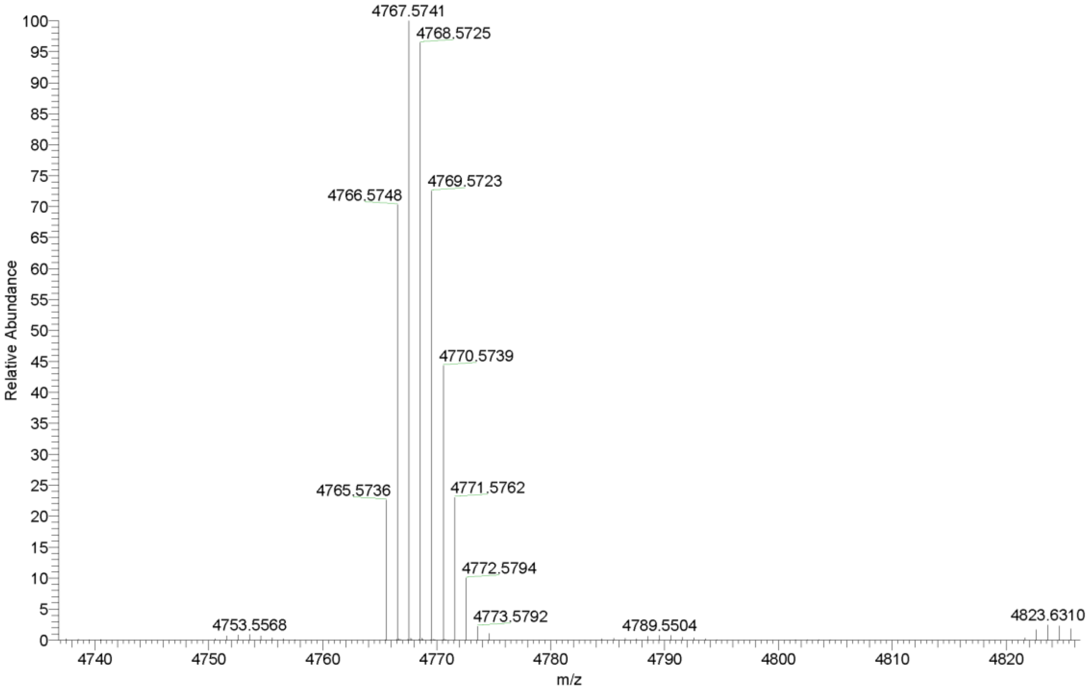
4767.5741
4768.5725
4766.5748
4769.5723
4770.5739
4765.5736
4771.5762
4772.5794
4773.5792
4753.5568
4789.5504
4823.6310
Relative Abundance
m/z

XC3 ((Ac-KL)$_8$(*K*KL)$_4$(*K*LL)$_2$*K*KL) was obtained from TentaGel S RAM resin (363.6 mg, 0.08 mmol, 0.22 mmol·g^{-1}), the dendrimer was obtained as a white foamy solid after preparative RP-HPLC purification (123.5 mg, 23.5%). Analytical RP-HPLC: t_R = 1.63 min (100% A to 100% B in 3.5 min, λ= 214 nm). MS (ESI+): $C_{238}H_{448}N_{60}O_{45}$ calc./obs. 4867.46/4867.49[M]$^+$.

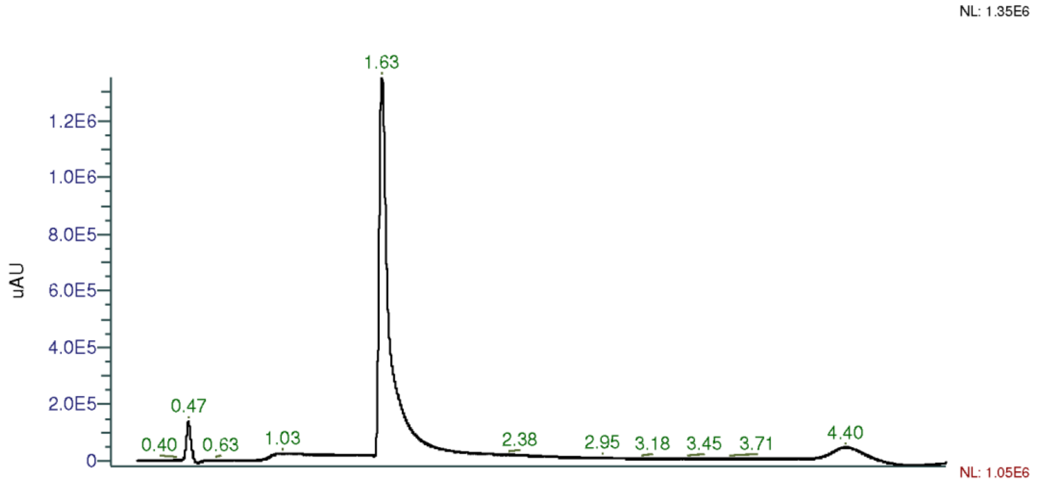

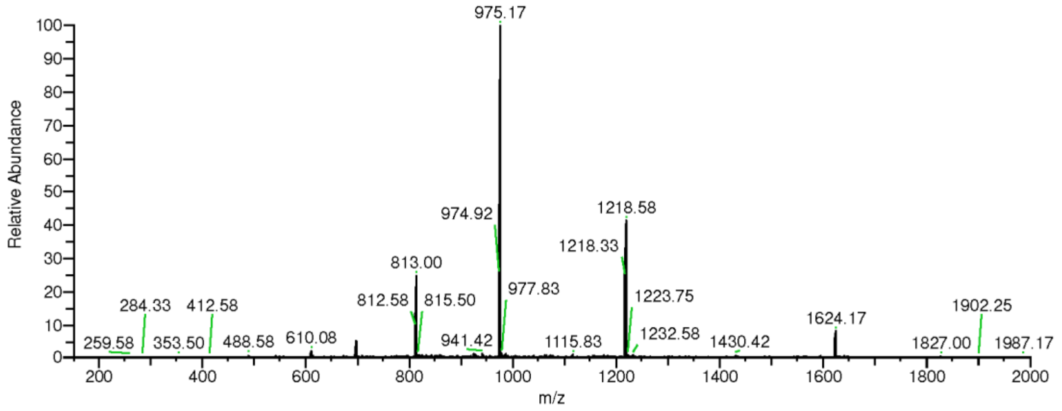

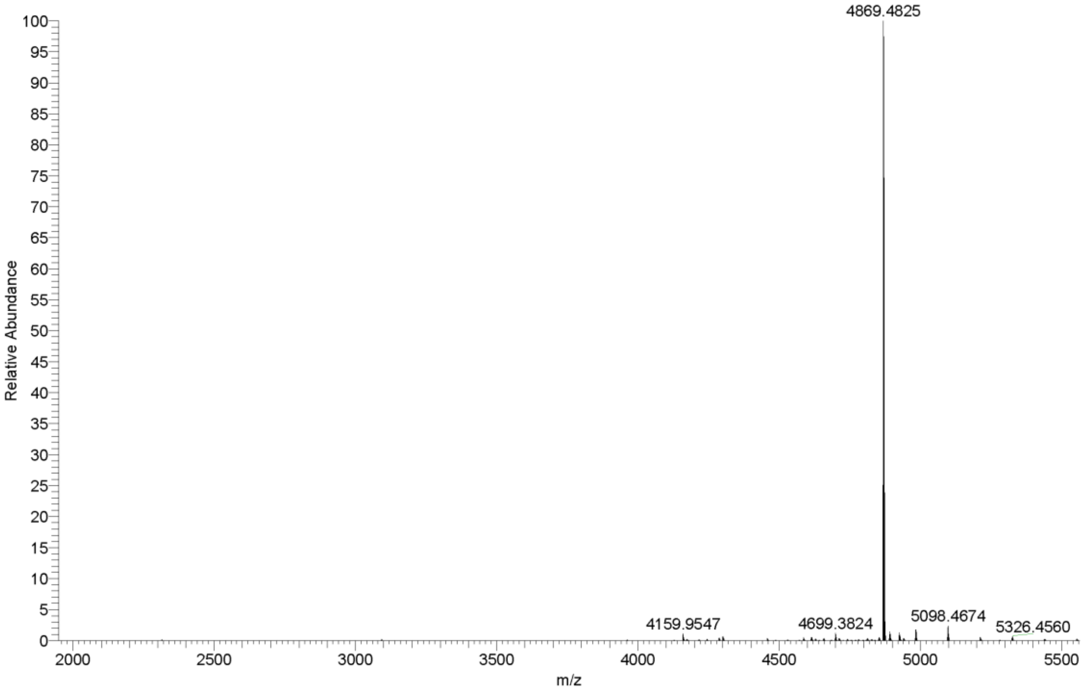
4869.4825
Relative Abundance
4159.9547
4699.3824
5098.4674
5326.4560
m/z

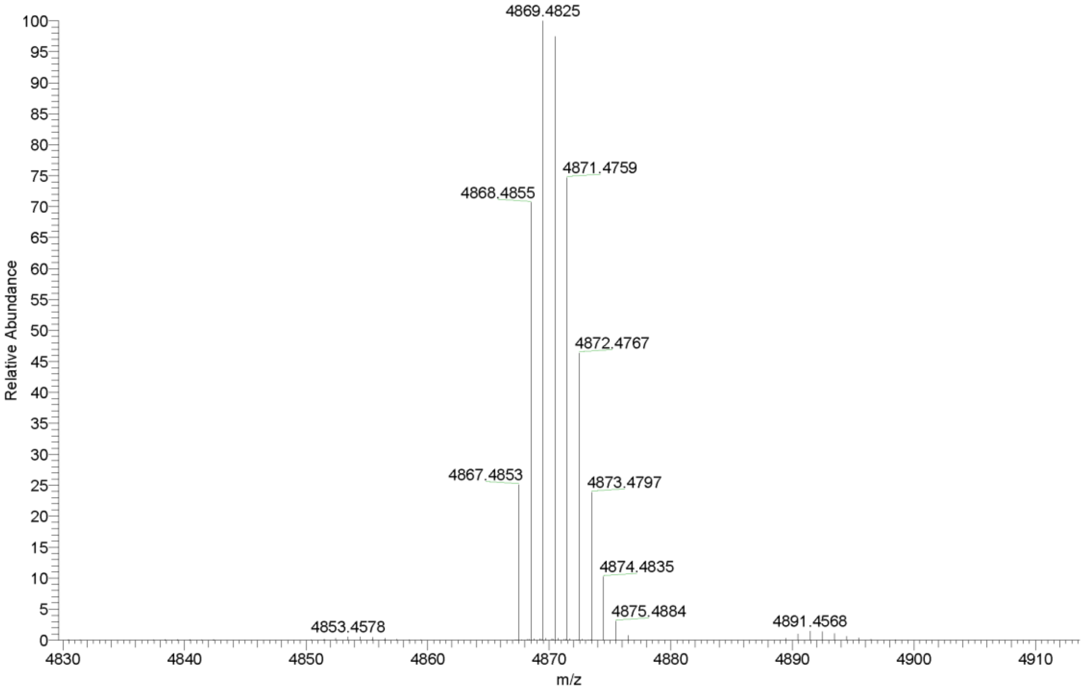
4869.4825
4871.4759
4868.4855
4872.4767
4867.4853
4873.4797
4874.4835
4875.4884
4853.4578
4891.4568
Relative Abundance
m/z

XC4 ((Ac-KL)$_8$(*K*KL)$_4$(*K*KLL)$_2$*K*KKL) was obtained from TentaGel S RAM resin (363.6 mg, 0.08 mmol, 0.22 mmol·g^{-1}), the dendrimer was obtained as a white foamy solid after preparative RP-HPLC purification (85.7 mg, 15.2%). Analytical RP-HPLC: t_R = 1.74 min (100% A to 100% B in 3.5 min, λ= 214 nm). MS (ESI+): $C_{256}H_{482}N_{64}O_{48}$ calc./obs. 5221.72/5221.75 [M]$^+$.

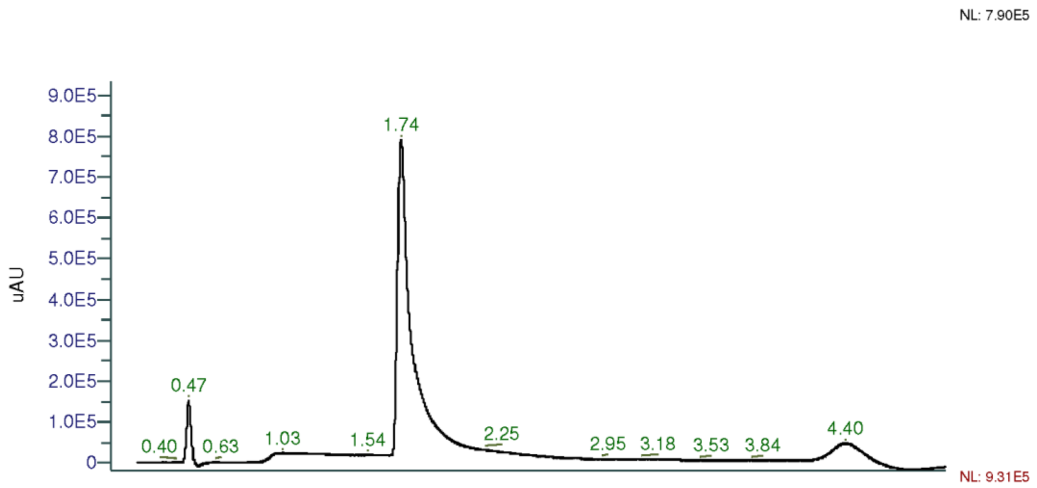

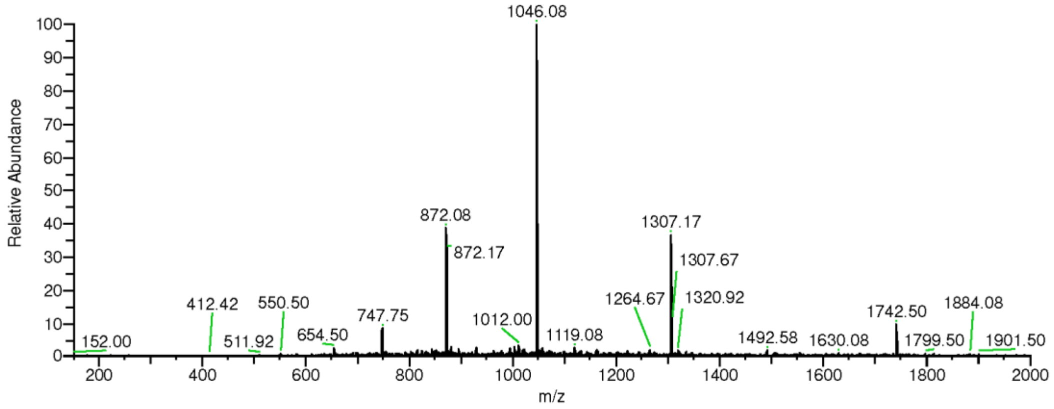

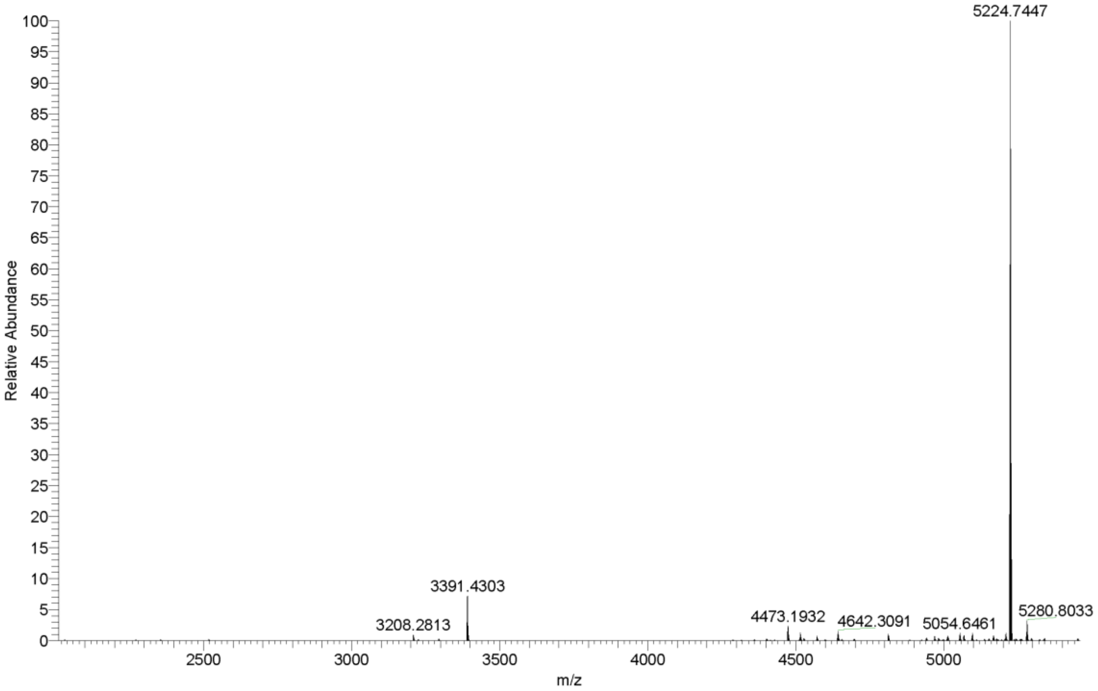
5224.7447
3391.4303
3208.2813
4473.1932
4642.3091
5054.6461
5280.8033
Relative Abundance
m/z

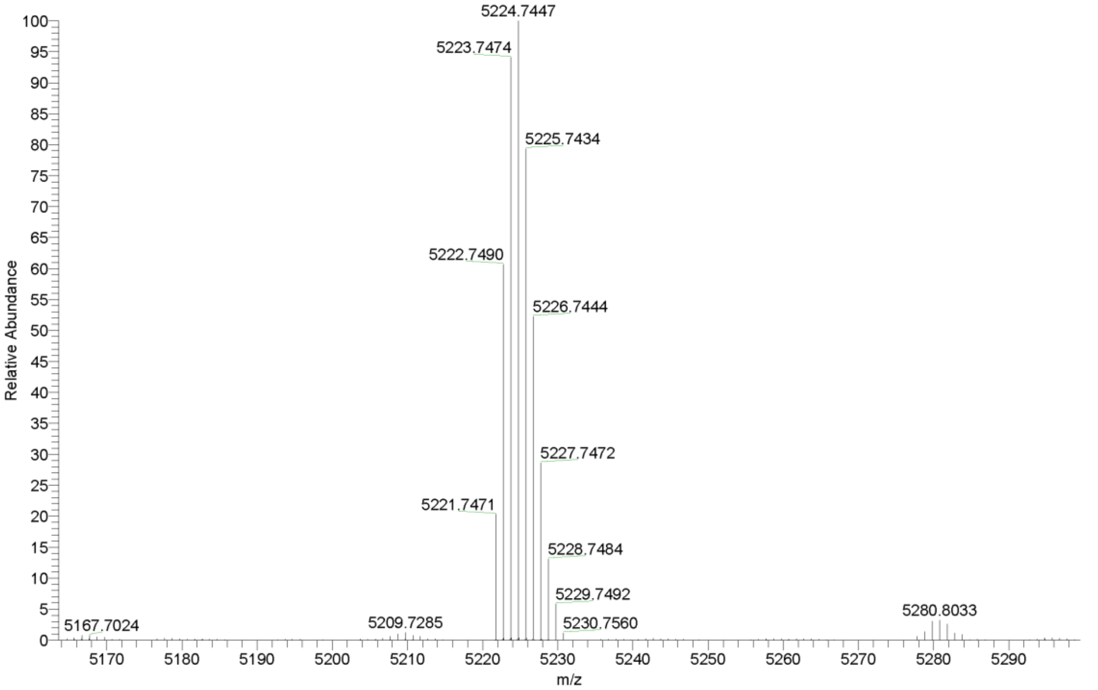
5224.7447
5223.7474
5225.7434
5222.7490
5226.7444
5227.7472
5221.7471
5228.7484
5229.7492
5230.7560
5280.8033
5209.7285
5167.7024
Relative Abundance
m/z

G3KL $((KL)_8(\mathit{K}KL)_4(\mathit{K}LL)_2\mathit{K}KL)$ was obtained from TentaGel S RAM resin (363.6 mg, 0.08 mmol, 0.22 mmol·g^{-1}), the dendrimer was obtained as a white foamy solid after preparative RP-HPLC purification (66.5 mg, 18.3%). Analytical RP-HPLC: t_R = 1.45 min (100% A to 100% B in 3.5 min, λ= 214 nm). MS (ESI+): $C_{238}H_{448}N_{60}O_{45}$ calc./obs. 4531.38/4531.37 $[M]^+$.

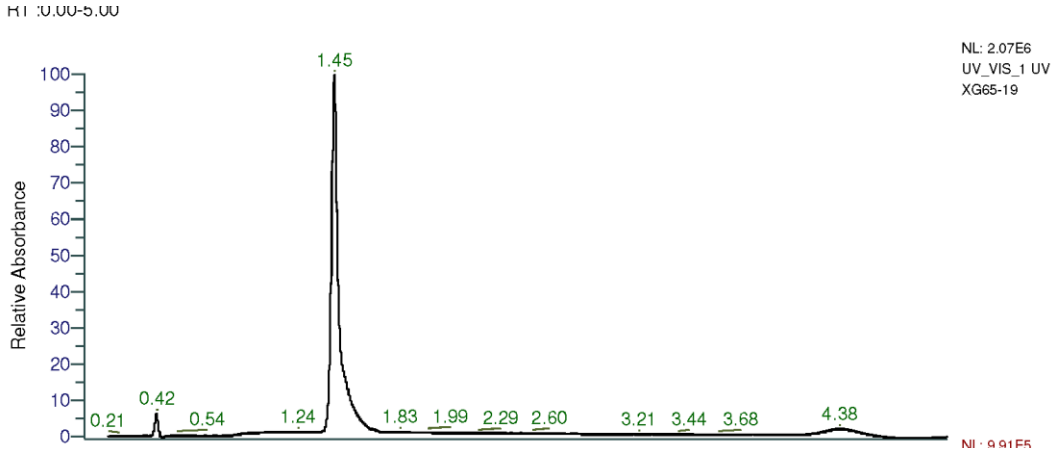

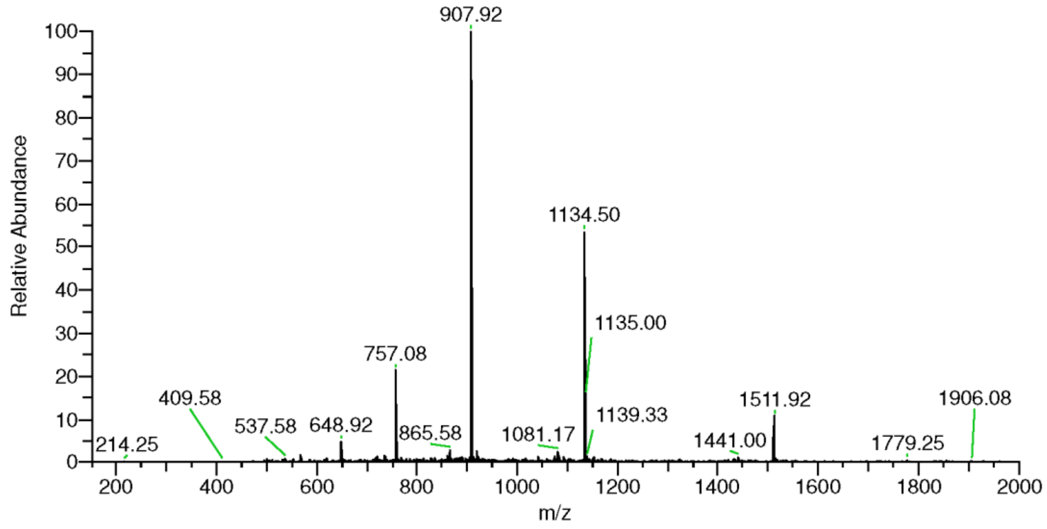

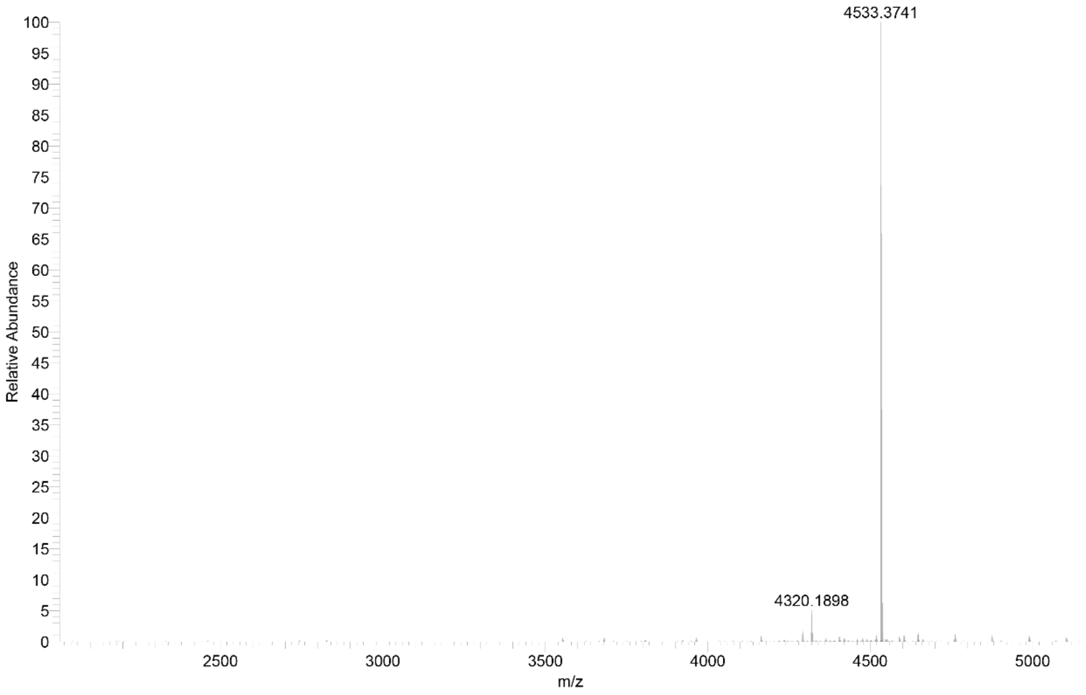
4533.3741
4320.1898
Relative Abundance
m/z
2500
3000
3500
4000
4500
5000

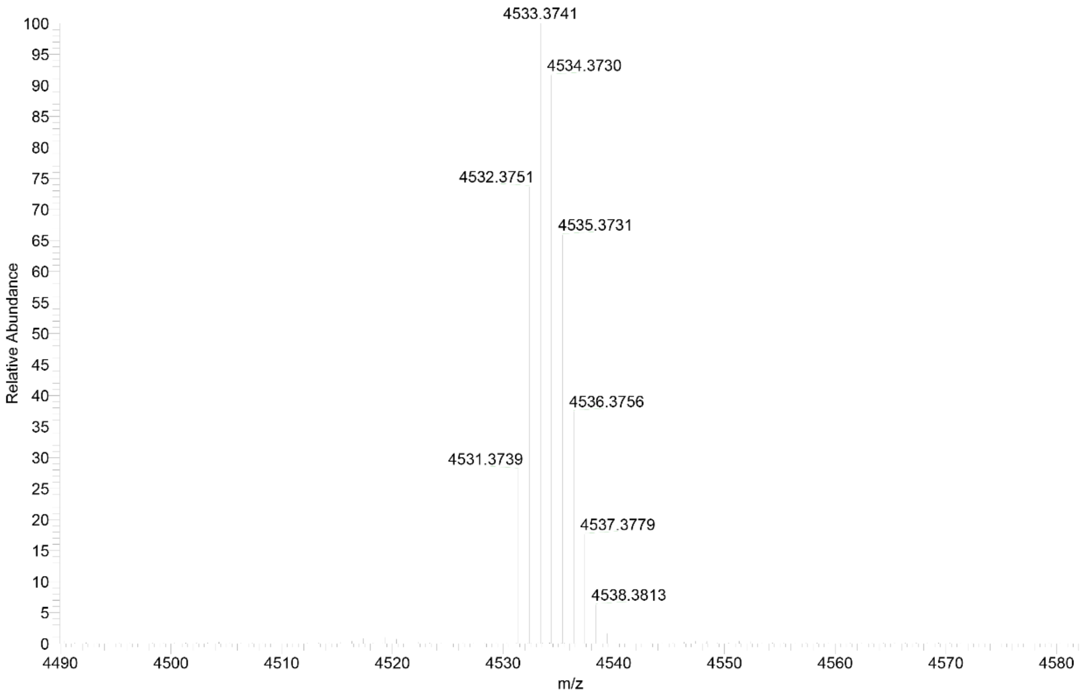
4533.3741
4534.3730
4532.3751
4535.3731
4536.3756
4531.3739
4537.3779
4538.3813
Relative Abundance
m/z

T7 ((KL)$_8$(*K*KL)$_4$(*K*KLL)$_2$*K*KKL) was obtained from TentaGel S RAM resin (363.6 mg, 0.08 mmol, 0.22 mmol·g^{-1}), the dendrimer was obtained as a white foamy solid after preparative RP-HPLC purification (118.7 mg, 19.5%). Analytical RP-HPLC: t_R = 1.59 min (100% A to 100% B in 3.5 min, λ= 214 nm). MS (ESI+): $C_{240}H_{466}N_{64}O_{40}$ calc./obs. 4885.64/4885.71 $[M]^+$.

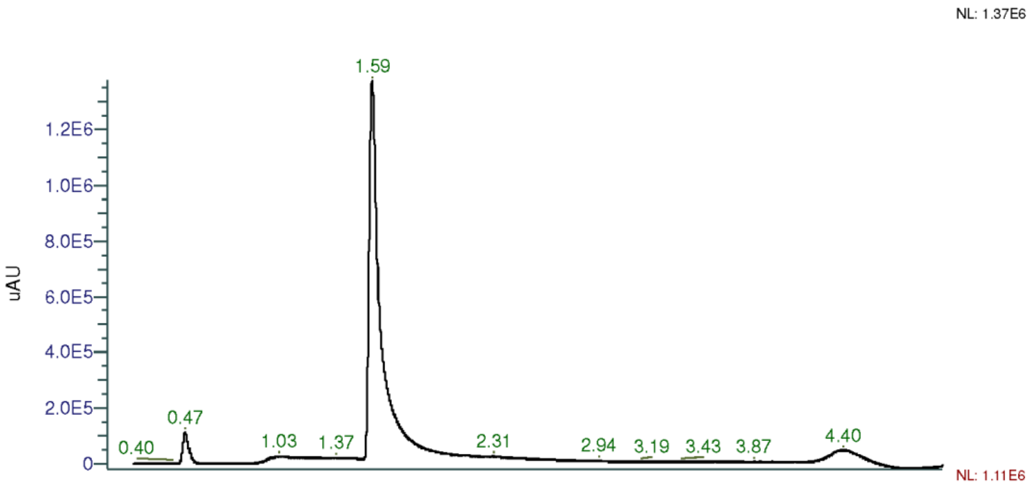

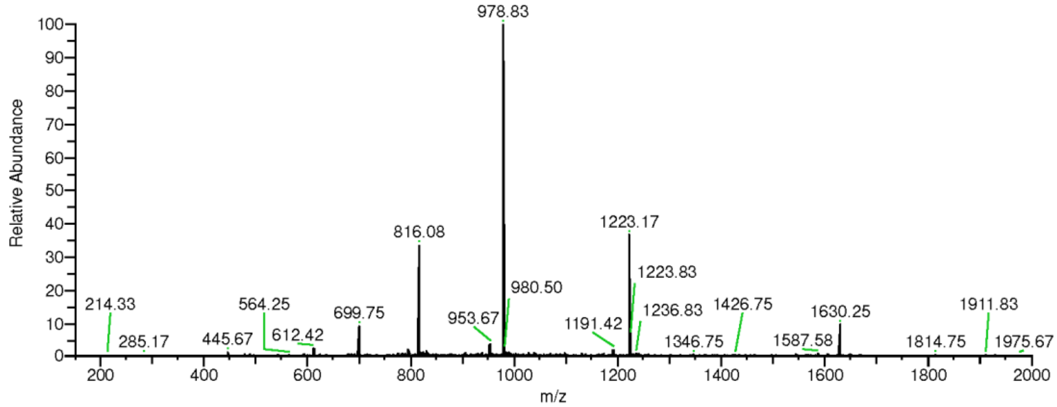

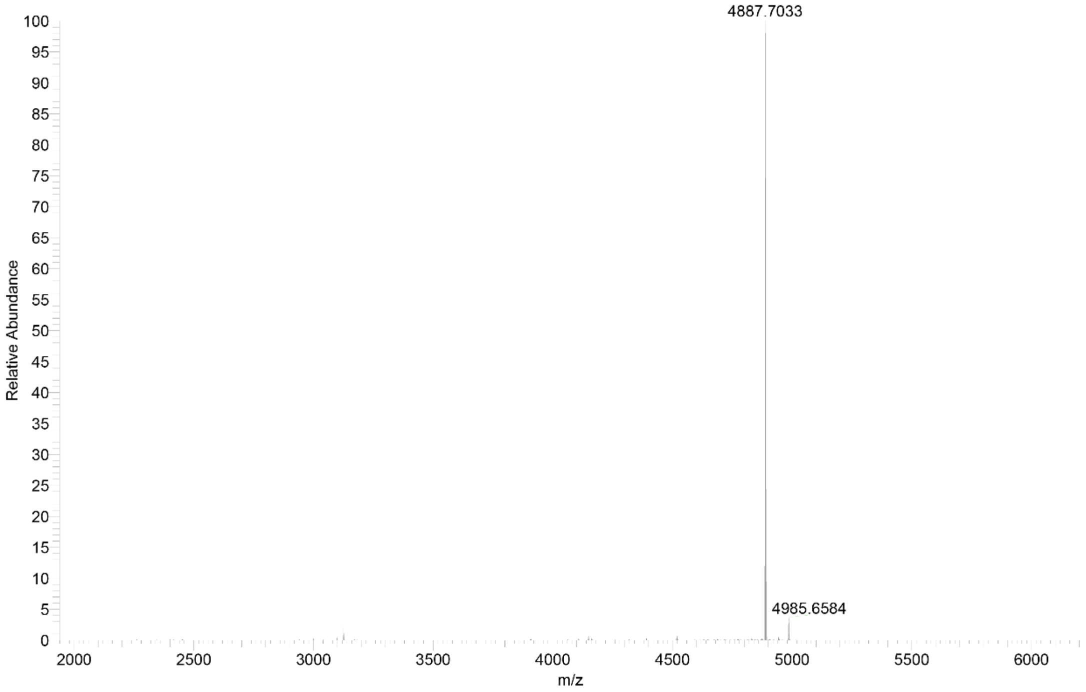
4887.7033
4985.6584
Relative Abundance
m/z

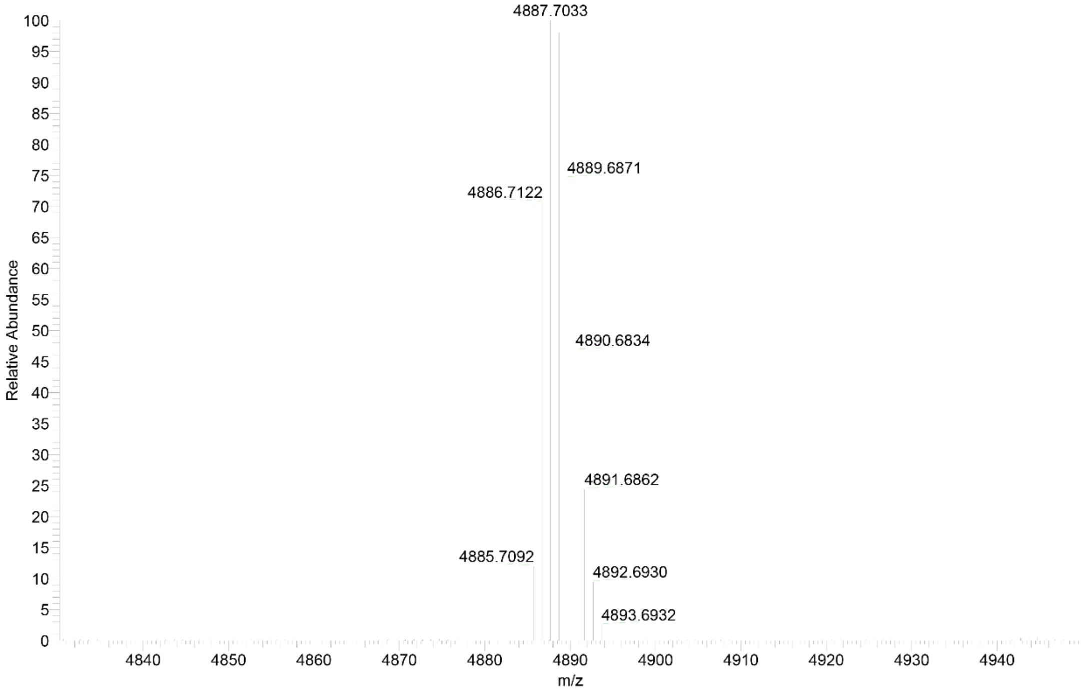
4887.7033
4889.6871
4886.7122
4890.6834
4891.6862
4885.7092
4892.6930
4893.6932
Relative Abundance
m/z

2. Acid-base titration

Powder peptide samples (13.00-16.00 mg) were diluted in Milli-Q water 10.0 mL (final concentration of dendrimers is 1.00 mg/mL) and acidified to pH ~3 with 1 M HCl. Then, 0.1 M NaOH was added in step of 2 μL with a Dosimat plus (Metrohm, Zofingen, Switzerland) and pH was measured on a 692 pH/ion meter (Metrohm).

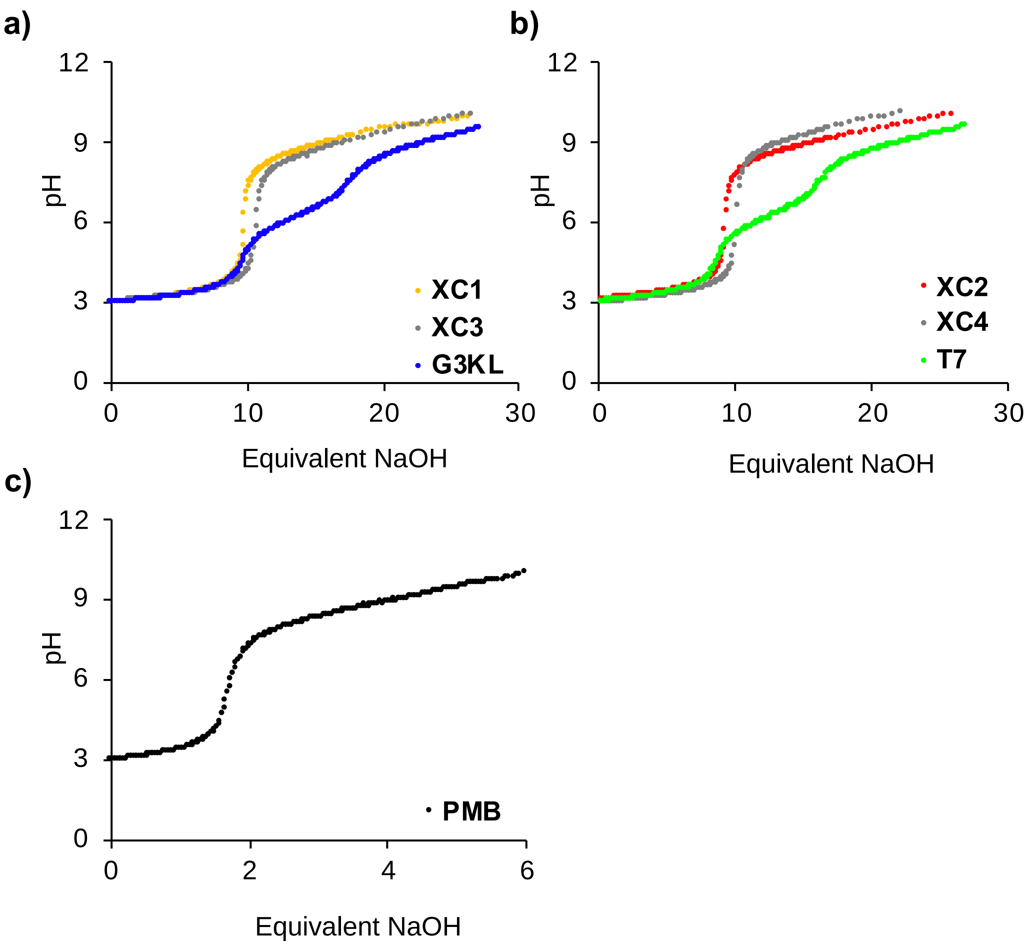

Fig. S1. Acid-base titration curves of **XC1, XC2, XC3, XC4, G3KL, T7** and **PMB**.

3. Circular dichroism (CD) spectroscopic measurements

CD spectra were recorded using a Jasco J-715 spectrometer equipped with a PFD-350S temperature controller and a PS-150J power supply. All experiments were measured using a Hellma Suprasil 100QS 0.1 cm cuvette. Stock solution (1.00 mg/mL) of dendrimers were freshly prepared in 10 mM phosphate buffer pH 7.4, 8.0 or acetate buffer pH 5.0. For the measurement, the peptides were diluted to 0.100 mg/mL with buffer. 5 mM dodecylphosphocholine (DPC, Avanti Polar Lipids, Inc., USA) or 10 mM Sodium dodecyl sulfate (SDS, Sigma Aldrich, Buchs, Switzerland) was added when specified. The range of measurement was 185-260 nm, scan rate was 20 nm/min, pitch 0.5 nm, response 16 sec. and band 1.0 nm. The nitrogen flow was kept above 10 L/min. The blank was recorded under the same conditions and subtracted manually. The cuvettes were washed with 1M HCl, mQ-H_2O and buffer before each measurement.

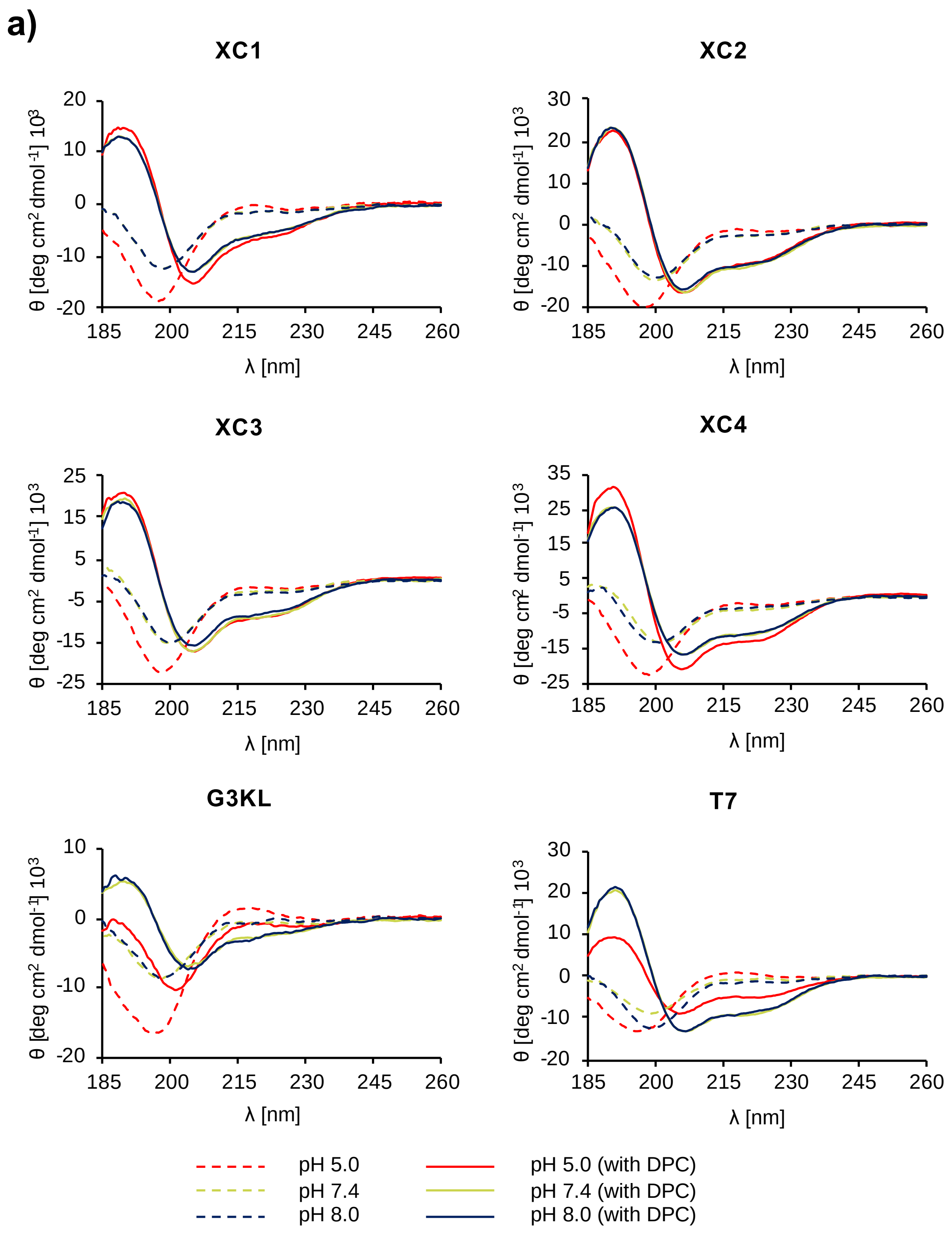

Fig. S2. CD spectra of **XC1, XC2, XC3, XC4, G3KL** and **T7** at different pH with or without 5 mM DPC. (pH 5.0: 10 mM acetate buffer, pH 7.4: 10 mM phosphate buffer, pH 8.0: 10 mM phosphate buffer.

b)

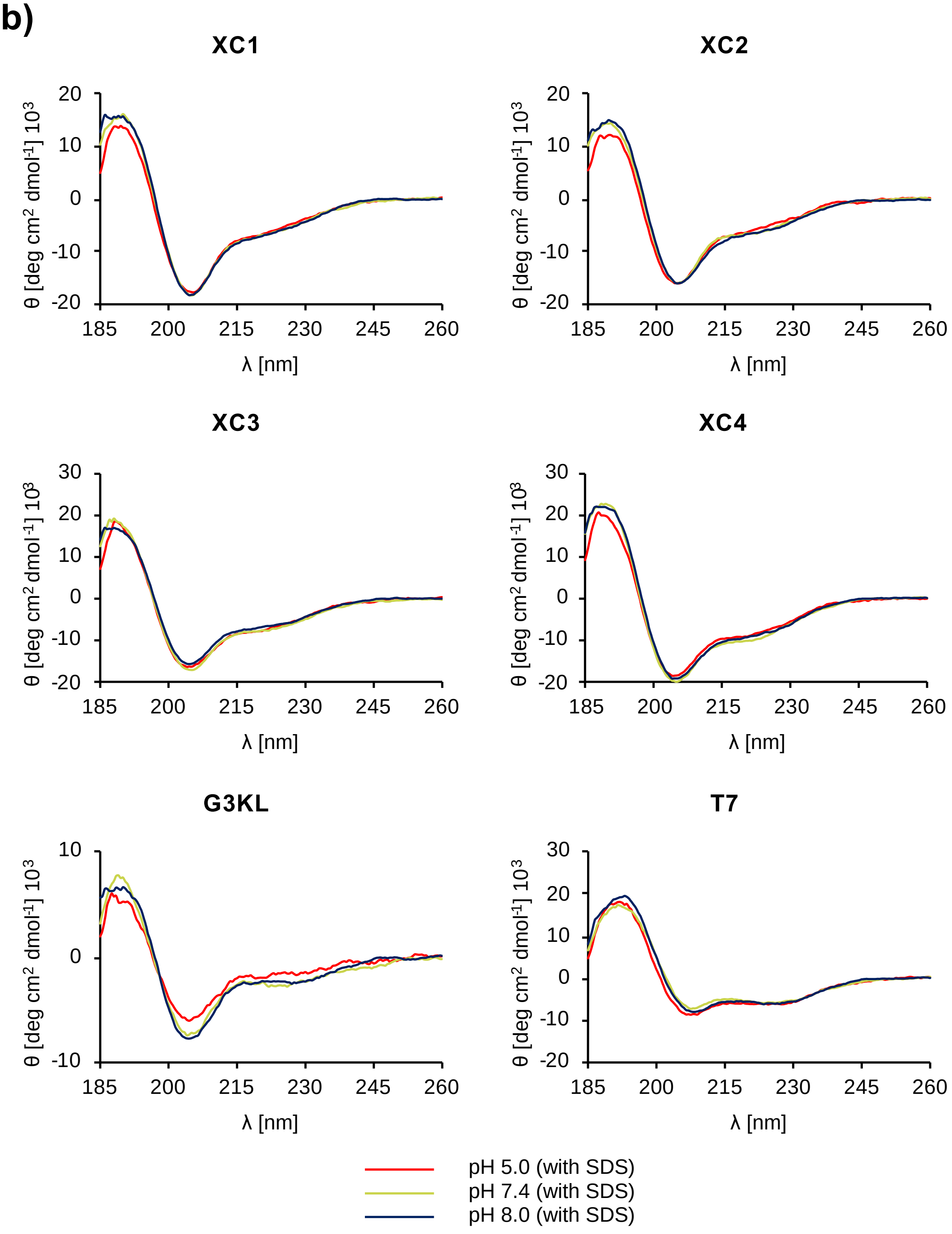

Fig. S3. CD spectra of **XC1, XC2, XC3, XC4, G3KL** and **T7** at different pH with 10 mM SDS. (pH 5.0: 10 mM acetate buffer, pH 7.4: 10 mM phosphate buffer, pH 8.0: 10 mM phosphate buffer.

4. Molecular Dynamics (MD)

MD simulations were performed for dendrimers **G3KL** and **XC1** using GROMACS software version 2020.4 and the gromos53a6 force field. The dendrimer topologies were built by combining topologies of two linear peptides with the same sequence, one with alpha and one with epsilon connectivity at the branching lysines, using in house scripts. The starting confirmation was built by hand in PyMol software by setting all the dihedral angles to α-helix conformation. A dodecahedral box was created around the peptide 1.0 nm from the edge of the system and filled with extended simple point charge water molecules. Sodium and chloride ions were added to produce an electroneutral solution at a final concentration of 0.15 M NaCl. The energy was minimized using a steepest gradient method to remove any close contacts before the system was subjected to a two-phase position-restrained MD equilibration procedure. The system was first allowed to evolve for 100 ps in a canonical NVT (N is the number of particles, V the system volume, and T the temperature) ensemble at 300 K before pressure coupling was switched on and the system was equilibrated for an additional 100 ps in the NPT (P is the system pressure) ensemble at 1.0 bar and used for production runs.

4.1 Parameters for the non-natural residue aminohexanoic acid (Ahx)

The parameters for the non-natural aminohexanoic acid were derived from LYSH residues of the Gromos53a6 force field and added to the aminoacids.rtp file. They were defined as follows:

```
[ AHX ] ; Derived from LYSH
 [ atoms ]
;    N     N    -0.31000     0
;    H     H     0.31000     0
   CA   CH2     0.00000     1 ; it's a CH2 now
   CB   CH2     0.00000     1
   CG   CH2     0.00000     2
   CD   CH2     0.00000     2
   CE   CH2     0.12700     3
   NZ    NL     0.12900     3
  HZ1     H     0.24800     3
  HZ2     H     0.24800     3
  HZ3     H     0.24800     3
    C     C       0.450     4
    O     O      -0.450     4
 [ bonds ]
;    N     H    gb_2
;    N    CA    gb_21
   CA    CB    gb_27
   CA     C    gb_27
   CB    CG    gb_27
   CG    CD    gb_27
   CD    CE    gb_27
   CE    NZ    gb_21
   NZ   HZ1    gb_2
   NZ   HZ2    gb_2
   NZ   HZ3    gb_2
    C     O    gb_5
    C    +N    gb_10
 [ angles ]
;  ai    aj    ak   gromos type
   -C     N     H     ga_32
   -C     N    CA     ga_31
    H     N    CA     ga_18
;    N    CA    CB     ga_13
;    N    CA     C     ga_13
   CB    CA     C     ga_13
   CA    CB    CG     ga_15
   CB    CG    CD     ga_15
   CG    CD    CE     ga_15
   CD    CE    NZ     ga_15
   CE    NZ   HZ1     ga_11
   CE    NZ   HZ2     ga_11
   CE    NZ   HZ3     ga_11
  HZ1    NZ   HZ2     ga_10
  HZ1    NZ   HZ3     ga_10
  HZ2    NZ   HZ3     ga_10
   CA     C     O     ga_30
   CA     C    +N     ga_19
    O     C    +N     ga_33
 [ impropers ]
;  ai    aj    ak    al   gromos type
;    N    -C    CA     H     gi_1
;   CA     N     C    CB     gi_2
    C    CA    +N     O     gi_1
 [ dihedrals ]
;  ai    aj    ak    al   gromos type
;  -CA    -C     N    CA     gd_14
;   -C     N    CA     C     gd_39
   CB    CA    C      O      gd_40
    C    CA    CB    CG     gd_34
;    N    CA     C    +N     gd_40
   CA    CB    CG    CD     gd_34
   CB    CG    CD    CE     gd_34
   CG    CD    CE    NZ     gd_34
   CD    CE    NZ   HZ1     gd_41   ;
```

4.2 MD in the presence of a DPC micelle

MD simulations in the presence of a DPC (dodecylphosphocholine) micelle were performed as follows. Parameters (itp for GROMOS53a6) and references for the DPC molecule are given below. Dendrimers were manually placed at a distance from the pre-equilibrated micelle (of 65 DPC molecules) approximatively equal to the diameter of said peptide. Box, solvation and NVT equilibration procedures were performed as explained above. For each peptide/micelle system, 10 runs of 100 ns were generated to show the possibility for the peptide to either interact or diffuse away from the micelle. Then, runs of interest where the dendrimer was interacting with the micelle, were extended to 1000 ns.

```
; Charge from Chiu et al.
; Chiu, S. W.; Clark, M.; Balaji, V.; Subramaniam, S.; Scott, H. L.; Jakobsson, E.
Incorporation of surface tension into molecular dynamics simulation of an interface: a fluid
phase lipid bilayer membrane. Biophys. J. 1995, 69, 1230-1245.
; Atom types from GROMOS53A6
; Oostenbrink, C.; Soares, T. A.; van der Vegt, N. F. A.; van Gunsteren, W. F. Validation of
the 53A6 GROMOS force field. Eur. Biophys. J. 2005, 34, 273-284.

[ moleculetype ]
; Name   nrexcl
DPC      3

[ atoms ]
;   nr    type   resnr  residu    atom    cgnr        charge          mass
     1     CH3       1    DPC      C1       1         0.40  15.035  ; qtot: 0.25
     2     CH3       1    DPC      C2       2         0.40  15.035  ; qtot: 0.50
     3     CH3       1    DPC      C3       3         0.40  15.035 ; qtot: 0.75
     4     NL        1    DPC      N4       4        -0.5  14.0067 ; qtot: 0.75
     5     CH2       1    DPC      C5       5         0.30  14.027 ; qtot:  1.0
     6     CH2       1    DPC      C6       6         0.40  14.027 ; qtot: 1.0
     7      OA       1    DPC      O7       7        -0.80  15.999 ; qtot: 0.64
     8       P       1    DPC      P8       8         1.7  30.973 ; qtot : 1.63
     9      OM       1    DPC      O9       9        -0.8 15.999 ; qtot: 0.995
    10      OM       1    DPC     O10      10        -0.8 15.999 ; qtot: 0.36
    11      OA       1    DPC     O11      11        -0.7  15.999 ; qtot: 0.0
    12     CH2       1    DPC     C12      12         0.0   14.027  ; qtot: 0
    13     CH2       1    DPC     C13      13         0.0   14.027  ; qtot: 0
    14     CH2       1    DPC     C14      14         0.0   14.027  ; qtot: 0
    15     CH2       1    DPC     C15      15         0.0   14.027  ; qtot: 0
    16     CH2       1    DPC     C16      16         0.0   14.027  ; qtot: 0
    17     CH2       1    DPC     C17      17         0.0   14.027  ; qtot: 0
    18     CH2       1    DPC     C18      18         0.0   14.027  ; qtot: 0
    19     CH2       1    DPC     C19      19         0.0   14.027  ; qtot: 0
    20     CH2       1    DPC     C20      20         0.0   14.027  ; qtot: 0
    21     CH2       1    DPC     C21      21         0.0   14.027  ; qtot: 0
    22     CH2       1    DPC     C22      22         0.0   14.027  ; qtot: 0
    23     CH3       1    DPC     C23      23         0.0   15.035  ; qtot: 0


[ bonds ]
;  ai    aj funct            c0            c1            c2            c3

    1     4     2    gb_21
    2     4     2    gb_21
    3     4     2    gb_21
    4     5     2    gb_21
    5     6     2    gb_27
    6     7     2    gb_18
    7     8     2    gb_28
    8     9     2    gb_24
    8    10     2    gb_24
    8    11     2    gb_28
```

```
   11    12     2    gb_18
   12    13     2    gb_27
   13    14     2    gb_27
   14    15     2    gb_27
   15    16     2    gb_27
   16    17     2    gb_27
   17    18     2    gb_27
   18    19     2    gb_27
   19    20     2    gb_27
   20    21     2    gb_27
   21    22     2    gb_27
   22    23     2    gb_27

[ pairs ]
;  ai    aj funct
    1     6     1
    2     6     1
    3     6     1
    4     7     1
    5     8     1
    6     9     1
    6    10     1
    6    11     1
    7    12     1
    8    13     1
    9    12     1
   10    12     1
   11    14     1
;   12    15     1
;   13    16     1
;   14    17     1
;   15    18     1
;   16    19     1
;   17    20     1
;   18    21     1
;   19    22     1
;   20    23     1

[ angles ]
;  ai    aj    ak funct
    1     4     2     2    ga_13
    1     4     3     2    ga_13
    1     4     5     2    ga_13
    2     4     3     2    ga_13
    2     4     5     2    ga_13
    3     4     5     2    ga_13
    4     5     6     2    ga_15
    5     6     7     2    ga_15
    6     7     8     2    ga_26
    7     8     9     2    ga_14
    7     8    10     2    ga_14
    7     8    11     2    ga_5
    9     8    10     2    ga_29
   10     8    11     1    ga_14
    8    11    12     1    ga_26
   11    12    13     1    ga_15
   12    13    14     1    ga_15
   13    14    15     1    ga_15
   14    15    16     1    ga_15
   15    16    17     1    ga_15
   16    17    18     1    ga_15
   17    18    19     1    ga_15
   18    19    20     1    ga_15
   19    20    21     1    ga_15
   20    21    22     1    ga_15
   21    22    23     1    ga_15

[ dihedrals ]
;  ai    aj    ak    al funct
    1     4     5     6     1 gd_29
    4     5     6     7     1 gd_4
    4     5     6     7     1 gd_36
    5     6     7     8     1 gd_29
;
; define gd_20     0.000       5.09          2
; O-P-O- (dna, lipids)  1.2
```

6	7	8	9	1	gd_20
7	8	11	12	1	gd_27
8	11	12	13	1	gd_29
11	12	13	14	1	gd_1
12	13	14	15	1	gd_34
13	14	15	16	1	gd_34
14	15	16	17	1	gd_34
15	16	17	18	1	gd_34
16	17	18	19	1	gd_34
17	18	19	20	1	gd_34
18	19	20	21	1	gd_34
19	20	21	22	1	gd_34
20	21	22	23	1	gd_34

5. MIC determination with different pH values and ionic strength

Mueller-Hinton (MH) medium was prepared at different pH. MH broth (Sigma Aldrich, Steinheim, Germany) was dissolved in 1 L of mQ water, adjust with 1 M NaOH or 1 M HCl until final pH is 5.0, 7.4 or 8.0. 0.1 M NaOH and 0.1 M HCl were used for precise adjustments. NaCl, KCl, $MgCl_2$ and $CaCl_2$ was added in the different concentration when specified. Medium was sterilized by autoclaving at 121 °C for 15 minutes.

Antimicrobial activity was assayed against *E. coli* W3110, *Acinetobacter baumannii* (ACTT 19606), *P. aeruginosa* PAO1 (WT), *K. pneumoniae* (NCTC 418), methicillin-resistant *Staphylococcus aureus* (COL). To determine MIC, broth microdilution method was used. A colony of bacteria was grown in LB (Lysogeny broth) medium overnight at 37 °C. The compounds were prepared as stock solutions of 8 mg/mL in mQ H_2O, diluted to the initial concentration of 64 µg/mL in 300 µL MH medium, added to the first well of 96-well microtiter plate (TPP, untreated) and diluted serially by ½. The concentration of the bacteria was quantified by measuring OD_{600} and diluted to OD_{600} = 0.022 in MH medium. The sample solutions (150 µL) were mixed with 4 µL diluted bacterial suspension with a final inoculation of about of 5 x 10^5 CFU. The plates were incubated at 37 °C until satisfactory growth (~18 h). For each test, two columns of the plate were kept for sterility control (broth only) and growth control (broth with bacterial, no antibiotics). The MIC was defined as the lowest concentration of the peptide dendrimer that inhibited visible growth of the tested bacteria, as detected after treatment with MTT. The assay was performed in the biosafety level 2 lab and was repeated at least two times.

Table S1. Influence of NaCl, KCl, $MgCl_2$, and $CaCl_2$ on MICs of **XC1** and **PMB** for *K. pneumoniae* and MRSA at pH 8.0.[a)]

	K. pneumoniae	MRSA	
	**XC1**	**XC1**	**PMB**
100 mM NaCl	8	32	16
200 mM NaCl	32	>64	32
300 mM NaCl	>64	>64	64
100 mM KCl	32	32	16
200 mM KCl	>64	>64	32
300 mM KCl	>64	>64	64
1 mM $MgCl_2$	8	8	8
3 mM $MgCl_2$	16	8	8
5 mM $MgCl_2$	32	8	8
1 mM $CaCl_2$	16	4	8
3 mM $CaCl_2$	>64	64	8
5 mM $CaCl_2$	>64	>64	8

[a)] MIC in µg/mL, measured in Müller–Hinton (MH) medium at pH 8.0 with additional salt on *K. pneumoniae* and MRSA after incubation for 16–20 h at 37 °C.

6. Relative antibiotics

Azithromycin, trimethoprim, vancomycin and novobiocin were purchased from Sigma Aldrich, erythromycin and ciprofloxacin was purchased from Acros Organics, spectinomycin was purchased from AppliChem.

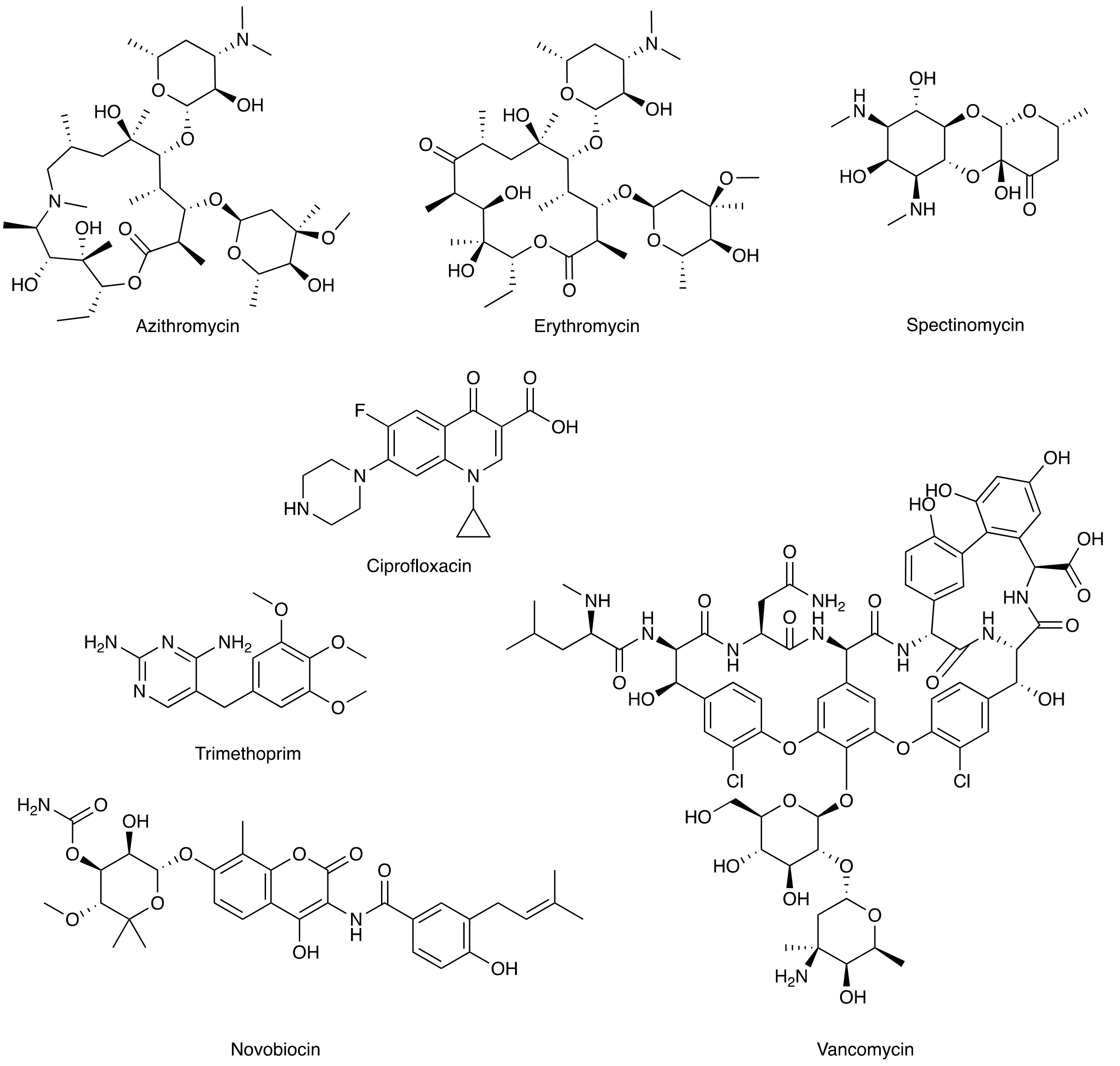

Fig. S4. Structures of different antibiotics used as control compounds.

Table S2. pH dependent antimicrobial activities (MIC at pH 5.0/pH 7.4/pH 8.6) of selected antibiotics [a)]

Cpd	*E. coli*	*A. baumannii*	*P. aeruginosa*	*K. pneumoniae*	MRSA	pK_a [b)]
azithromycin	>32/8/2	>32/16/0.25	>32/16/1	>32/2/0.25	>32/2/0.01	8.50
erythromycin	>32/32/2	>32/16/2	>32/32/16	>32/32/4	>32/<0.25/<0.25	8.88
spectinomycin	>32/8/1	>32/>32/32	>32/>32/32	>32/16/2	>32/>32/8	6.95
ciprofloxacin	4/0.25/0.5	4/1/0.5	0.5/<0.25/<0.25	1/<0.25/<0.25	2/0.125/0.125	6.09
trimethoprim	>32/<0.25/2	>32/>32/32	>32/>32/>32	>32/>32/>32	>32/>32/>32	7.12
vancomycin	>32/>32/>32	>32/>32/>32	>32/>32/>32	>32/>32/>32	1/0.5/2	7.75
novobiocin	4/>32/>32	<0.25/4/32	8/>32/>32	1/16/>32	<0.25/<0.25/4	4.30

a) MIC = minimal inhibitory concentration in µg/mL, measured in Müller–Hinton (MH) medium at pH 5.0/7.4/8.6 on *E. coli*, *A. baumannii*, *P. aeruginosa* PAO1, *K. pneumoniae* and MRSA after incubation for 16–20 h at 37 °C.
b) Experimental pK_a data from go.drugbank.com

7. Hemolysis assay

Compounds were subjected to a hemolysis assay to assess the hemolytic effect on human red blood cells (hRBCs). The blood was obtained from Interregionale Blutspende SRK AG, Bern, Switzerland. 1.5 mL of whole blood was centrifuged at 3000 rpm for 15 minutes at 4 °C. The plasma was discarded, and the hRBC pellet was re-suspended in 5 mL of PBS (pH 7.4) then centrifuged at 3000 rpm for 5 minutes at 4 °C. The washing of hRBC was repeated three times and the remaining pellet was re-suspended in 10 mL of PBS.

The samples were prepared as the initial concentration of 4000 µg/mL in PBS, added to the first well of 96-well microtiter plate (TPP, untreated) and diluted serially by ½. After diluted, 100 µL of sample was in each well and the final sample concentration was 4000 µg/mL, 2000 µg/mL, 1000 µg/mL, 500 µg/mL, 250 µg/mL, 125 µg/mL, 62.5 µg/mL and 31.3 µg/mL. Controls on each plate included a blank medium control (PBS 100 µL) and a hemolytic activity control (0.1% Triton™ X-100). 100 µL of hRBC suspension was incubated with 100 µL of each sample in PBS in 96-well plate (Nunc 96-Well Polystyrene Conical Bottom MicroWell Plates). After the plates were incubated for 4 h at room temperature, minimal hemolytic concentration (MHC) was determined by visual inspection of the wells. 100 µL supernatants was carefully pipetted to a flat bottom, clear wells plate (TPP® tissue culture plates, polystyrene). Hemolysis was measured by analyzing the absorbance of free hemoglobin leaked out of compromised in the supernatants at 540 nm with a plate reader (Tecan instrument Infinite M1000). The percentage hemolysis was determined as:

$$hemolysis(\%) = \frac{A_{compounds} - A_{PBS}}{A_{0.1\%\,Triton} - A_{PBS}} \times 100\%.$$

The assay was performed in the biosafety level 2 lab and was repeated at least two times.

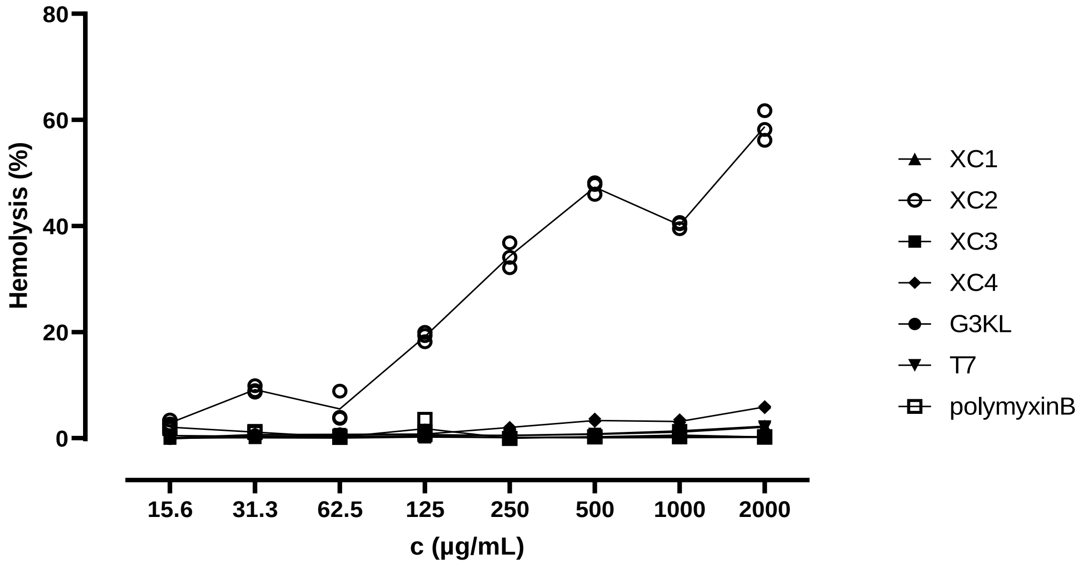

Fig. S5. Percentage of hemolysis of reference compounds

Table S3. Percentage of hemolysis of reference compounds

c (µg/mL)	**XC1**			**XC2**			**XC3**		
	Mean	SD	N	Mean	SD	N	Mean	SD	N
15.6	0.0	0.1	3	2.9	0.5	3	0.1	0.1	3
31.3	0.1	0.0	3	9.1	0.6	3	0.1	0.0	3
62.5	0.1	0.0	3	5.5	2.9	3	0.0	0.0	3
125	0.4	0.0	3	19.1	0.9	3	0.2	0.0	3
250	0.5	0.0	3	34.3	2.4	3	0.1	0.0	3
500	0.8	0.0	3	47.3	1.2	3	0.2	0.0	3
1000	1.4	0.1	3	40.2	0.6	3	0.3	0.0	3
2000	2.2	0.0	3	58.7	2.8	3	0.2	0.0	3

c (µg/mL)	**XC4**			**G3KL**			**T7**			**PMB**		
	Mean	SD	N	Mean	SD	N	Mean	SD	N	Mean	SD	N
15.6	0.1	0.1	3	-0.1	0.0	3	0.5	0.1	3	2.1	0.3	3
31.3	0.7	0.1	3	0.4	0.3	3	0.3	0.1	3	1.1	0.1	3
62.5	0.7	0.3	3	0.3	0.5	3	0.5	0.2	3	0.2	0.1	3
125	0.8	1.0	3	0.3	0.1	3	0.7	0.1	3	1.8	1.6	3
250	2.0	0.1	3	0.1	0.2	3	0.5	0.1	3	-0.1	0.0	3
500	3.3	0.3	3	0.0	0.0	3	0.7	0.1	3	0.2	0.0	3
1000	3.1	0.3	3	0.1	0.1	3	1.1	0.1	3	0.6	0.6	3
2000	5.9	0.1	3	0.1	0.0	3	2.1	0.2	3	0.2	0.1	3

8. Time kill kinetics assay

Time-kill kinetics was performed at pH 5.0 against *E. coli* (**XC1** 8 µg/mL, **G3KL** 128 µg/mL and **PMB** 0.08 µg/mL), *A. baumannii* (**XC1** 4 µg/mL, **G3KL** 32 µg/mL and **PMB** 4 µg/mL), *P. aeruginosa* PAO1 (**XC1** 32 µg/mL, **G3KL** 64 µg/mL and **PMB** 0.12 µg/mL), and *K. pneumoniae* (**XC1** 64 µg/mL, **G3KL** 64 µg/mL and **PMB** 32 µg/mL), at pH 7.4 and pH 8.0 against *K. pneumoniae* (**XC1** 8 µg/mL, **G3KL** 16 µg/mL and **PMB** 1 µg/mL) and MRSA (**XC1** 8 µg/mL, **G3KL** 8 µg/mL and **PMB** 16 µg/mL). Untreated bacteria at 1 x 10^6 CFU/mL was used as a growth control.

A single colony of bacteria was picked and grown overnight with shaking (180 rpm) in LB (Sigma Aldrich, Buchs, Switzerland) medium 5 mL overnight at 37 °C. The overnight bacterial culture was diluted to OD_{600} 0.002 (2 x 10^6 CFU/mL) in fresh MH (Sigma Aldrich, Buchs, Switzerland) medium. Stock solutions of **G3KL** and antibiotics in sterilized milliQ water were prepared in 1 mg/mL (**XC1**, **G3KL** and **PMB**) or 100 µg/mL (**PMB**) and were diluted to two times more than required concentration in fresh MH (Sigma Aldrich, Buchs, Switzerland) medium. 100 µL prepared bacteria solution in MH and 100 µL samples in MH were mixed in 96-well microtiter plate (TPP, untreated, Corning Incorporated, Kennebunk, USA). 96-well microtiter plates were incubated in 37 °C with shaking (180 rpm). Surviving bacteria were quantified at 0, 0.5, 1, 2, 3, 4, 5 and 6 hours by plating 10-fold dilutions of sample in sterilized normal saline on LB agar plates. LB agar plates were incubated at 37 °C for 10 hours and the number of individual colonies was counted at each time-point. The assay was performed in triplicate in the biosafety level 2 lab and repeated at least three times.

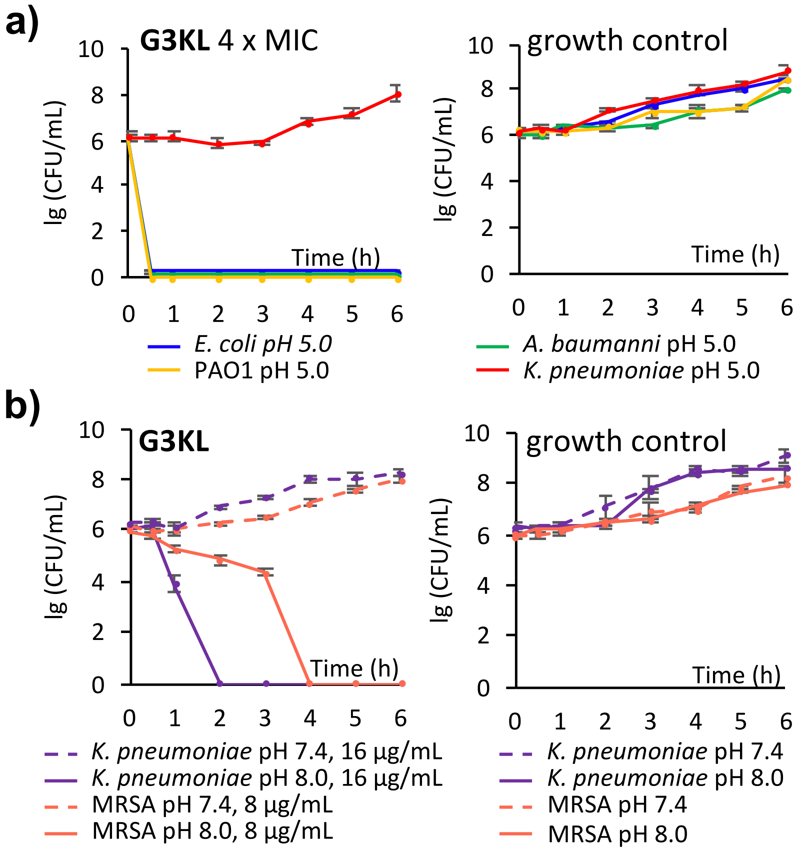

Fig. S6. a) Bacteria killing assay of **G3KL** at a concentration of 4 x MIC at pH 5.0 against *P. aeruginosa* PAO1, *A. baumannii*, *E. coli* and *K. pneumoniae* and growth control. b) Bacteria killing assay of **G3KL** at pH 7.4 and pH 8.0 against *K. pneumoniae* and MRSA.

9. Transmission electron microscopy (TEM)

Exponential phase (1 mL, OD_{600} = 1) of *Klebsiella pneumoniae* and MRSA were washed with MH medium and treated with **XC1**, **PMB** and **G3KL** in MH medium (at pH 7.4 or 8.0). Each time, 1 mL of the bacteria were centrifuged after 1 and 2 hours at 12 000 rpm for 3 min and fixed overnight with 2.5% glutaraldehyde (Agar Scientific, Stansted, Essex, UK) in 0.15 M HEPES (Fluka, Buchs, Switzerland) with an osmolarity of 670 mOsm and adjusted to a pH of 7.35. The next day, samples were washed with 0.15 M HEPES three times for 5 min, postfixed with 1% OsO_4 (SPI Supplies, West Chester, USA) in 0.1 M Na-cacodylate-buffer (Merck, Darmstadt, Germany) at 4 °C for 1 h. Thereafter, bacteria cells were washed in 0.1 M Na-cacodylate-buffer three times for 5 min and dehydrated in 70, 80, and 96% ethanol (Alcosuisse, Switzerland) for 15 min each at room temperature. Subsequently, they were immersed in 100% ethanol (Merck, Darmstadt, Germany) three times for 10 min, in acetone (Merck, Darmstadt, Germany) two times for 10 min, and finally in acetone-Epon (1:1) overnight at room temperature. The next day, bacteria cells were embedded in Epon (Fluka, Buchs, Switzerland) and hardened at 60 °C for 5 days.Sections were produced with an ultramicrotome UC6 (Leica Microsystems, Vienna, Austria), first semithin sections (1µm) for light microscopy which were stained with a solution of 0.5% toluidine blue O (Merck, Darmstadt, Germany) and then ultrathin sections (70-80 nm) for electron microscopy. The sections, mounted on single slot copper grids, were stained with 1% uranyl acetate at 40 °C for 30 min and 3% lead citrate at RT for 20 min or UranyLess (Electron Microscopy Sciences, Hatfield, UK) at 40 °C for 10 min and 3% lead citrate at 25 °C for 10 min with an ultrostainer (Leica Microsystems, Vienna, Austria). Sections were then examined with a Tecnai Spirit transmission electron microscope equipped with two digital cameras (FEI Eagle CCD Camera). The growth, incubation and fixation were performed in the biosafety level 2 lab.

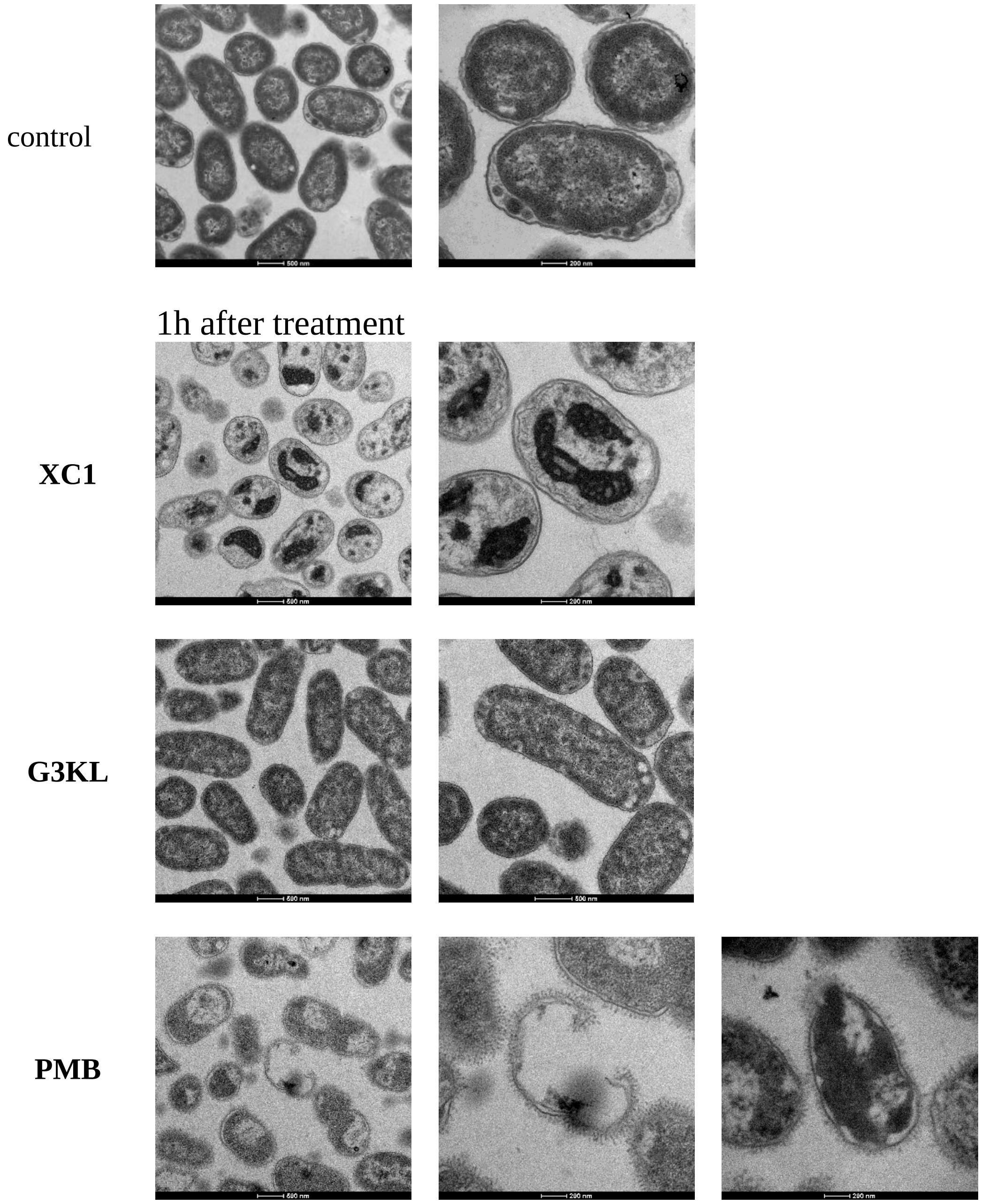

Fig. S7. TEM images of *K. pneumoniae*, 1 h after treatment with **XC1** (20 µg/mL), **G3KL** (40 µg/mL), and **PMB** (2.5 µg/mL) in MH medium at pH 7.4.

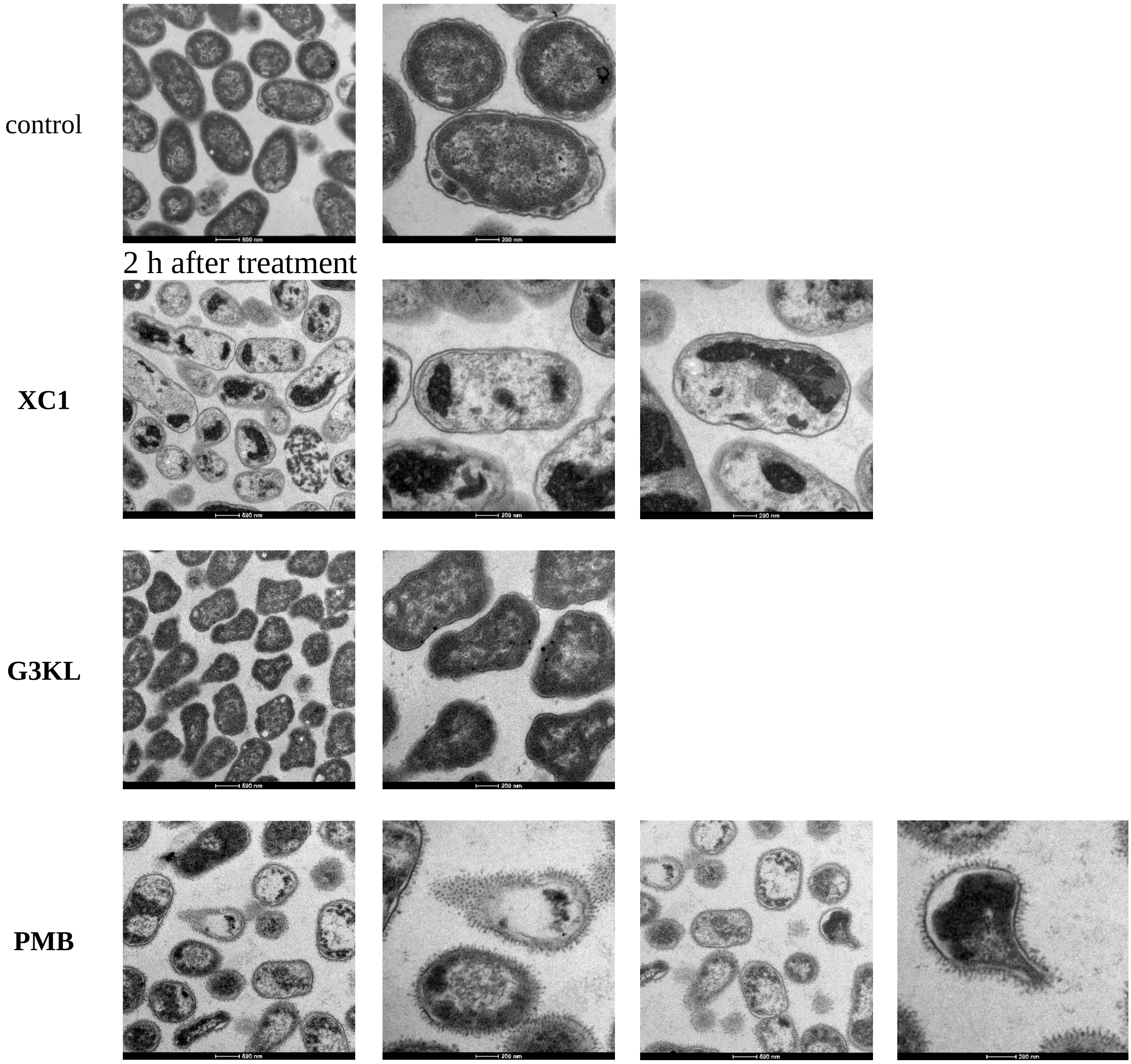

Fig. S8. TEM images of *K. pneumoniae*, 2 h after treatment with **XC1** (20 µg/mL), **G3KL** (40 µg/mL), and **PMB** (2.5 µg/mL) in MH medium at pH 7.4.

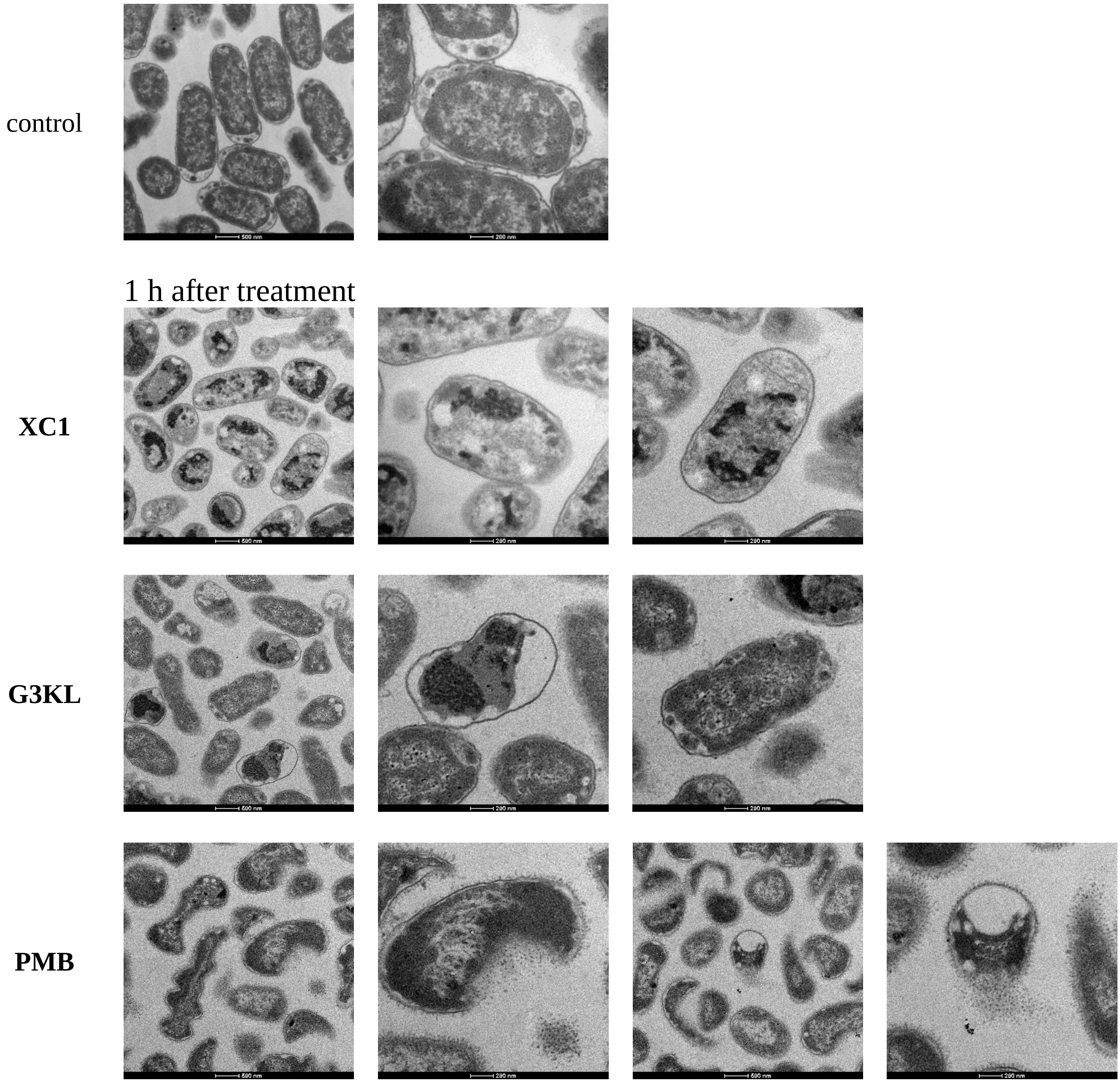

Fig. S9. TEM images of *K. pneumoniae*, 1 h after treatment with **XC1** (20 µg/mL), **G3KL** (40 µg/mL), and **PMB** (2.5 µg/mL) in MH medium at pH 8.0.

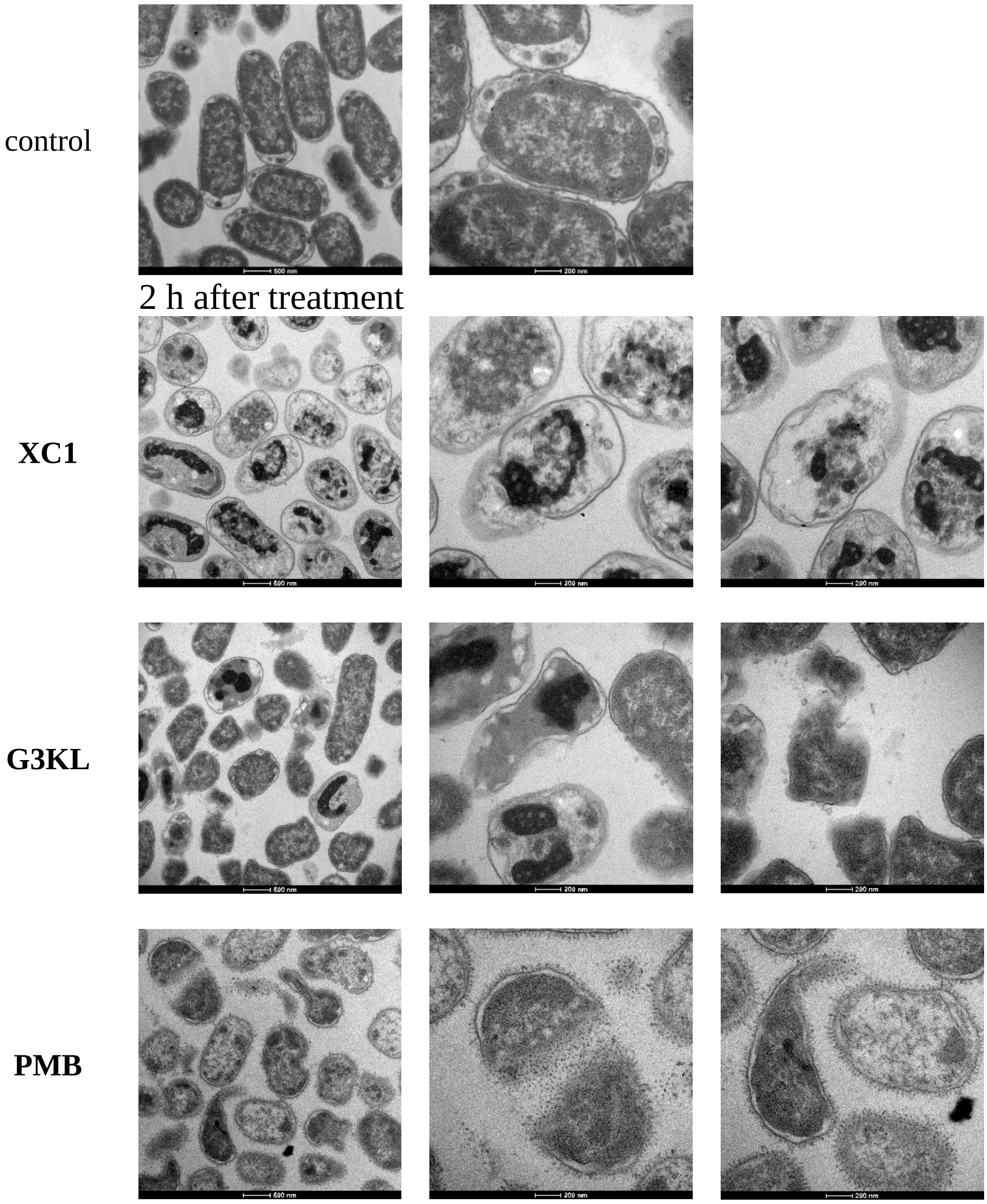

Fig. S10. TEM images of *K. pneumoniae*, 2 h after treatment with **XC1** (20 µg/mL), **G3KL** (40 µg/mL), and **PMB** (2.5 µg/mL) in MH medium at pH 8.0.

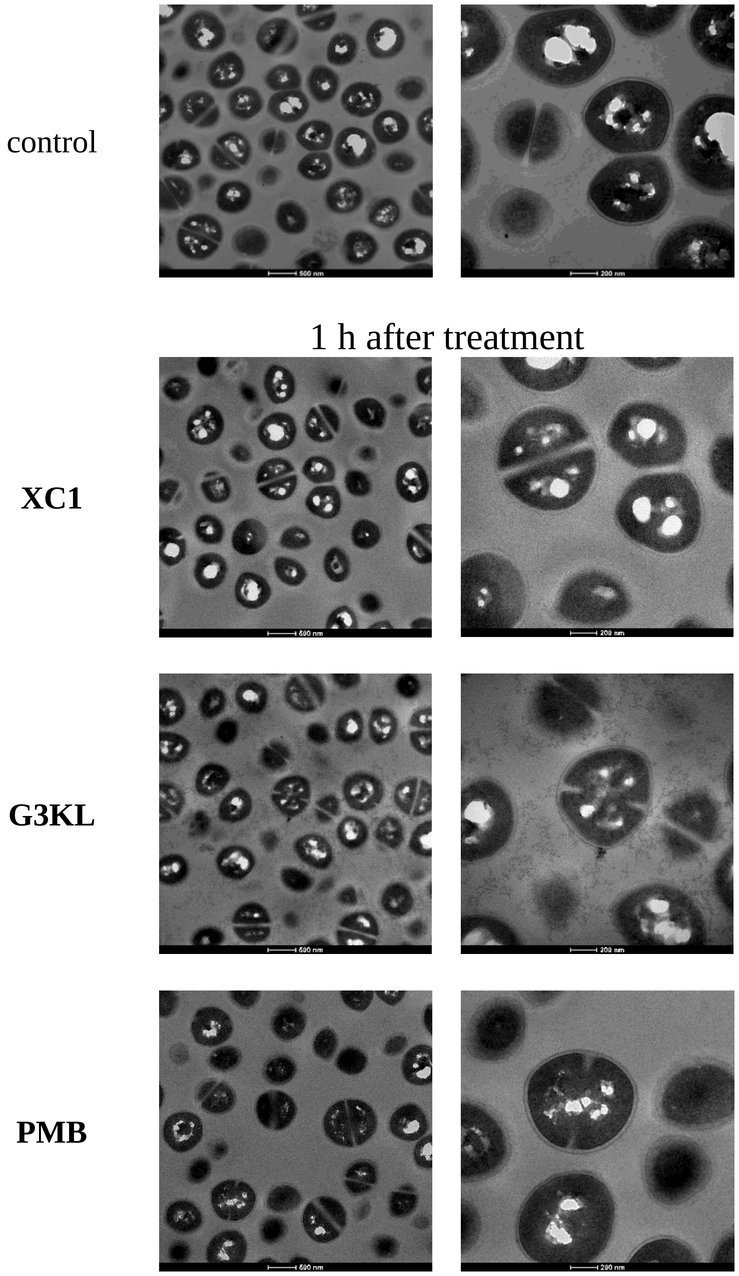

Fig. S11. TEM images of MRSA, 1 h after treatment with **XC1** (20 µg/mL), **G3KL** (20 µg/mL), and **PMB** (40 µg/mL) in MH medium at pH 7.4.

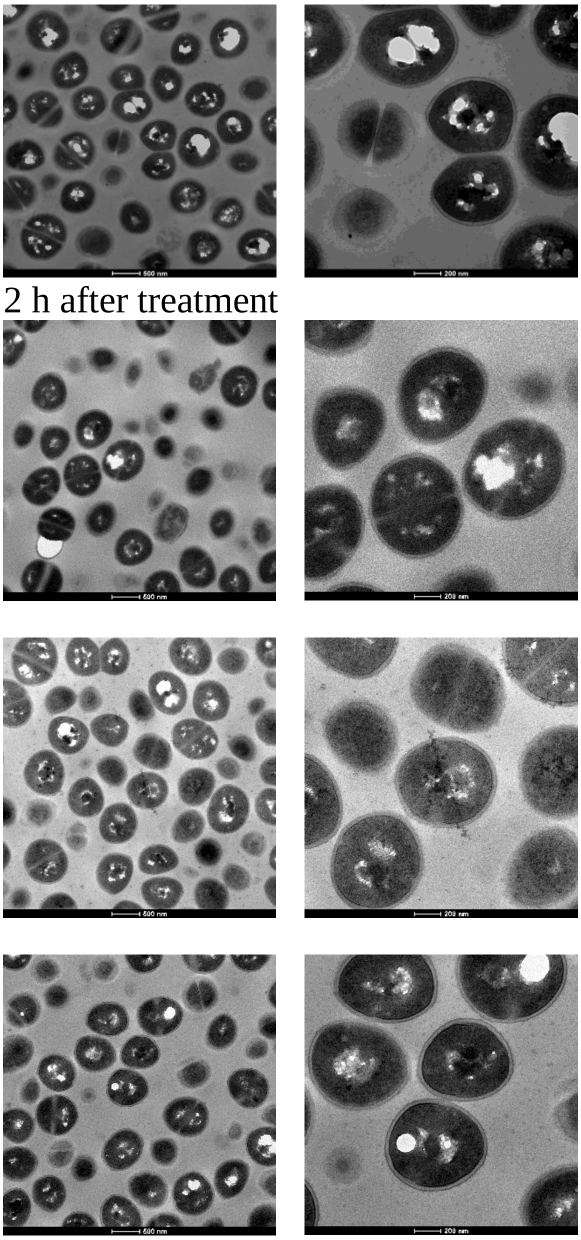

Fig. S12. TEM images of MRSA, 2 h after treatment with **XC1** (20 µg/mL), **G3KL** (20 µg/mL), and **PMB** (40 µg/mL) in MH medium at pH 7.4.

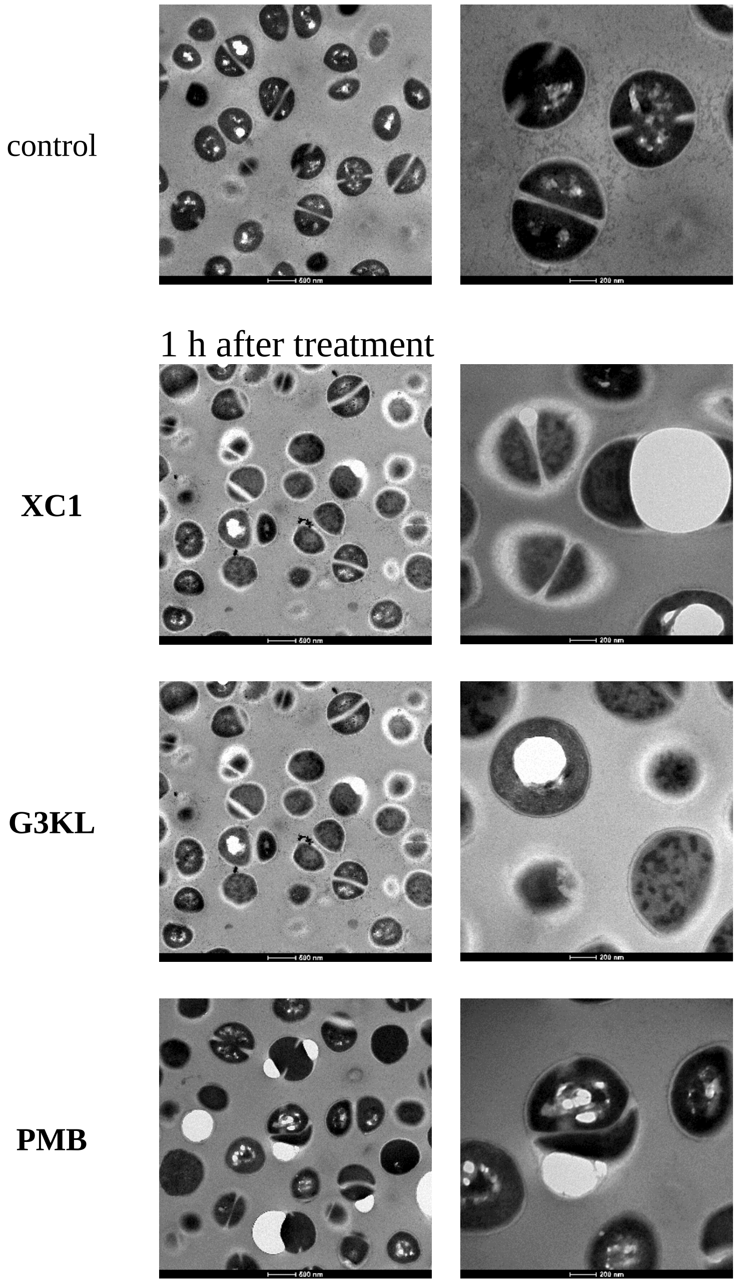

Fig. S13. TEM images of MRSA, 1 h after treatment with **XC1** (20 µg/mL), **G3KL** (20 µg/mL), and **PMB** (40 µg/mL) in MH medium at pH 8.0

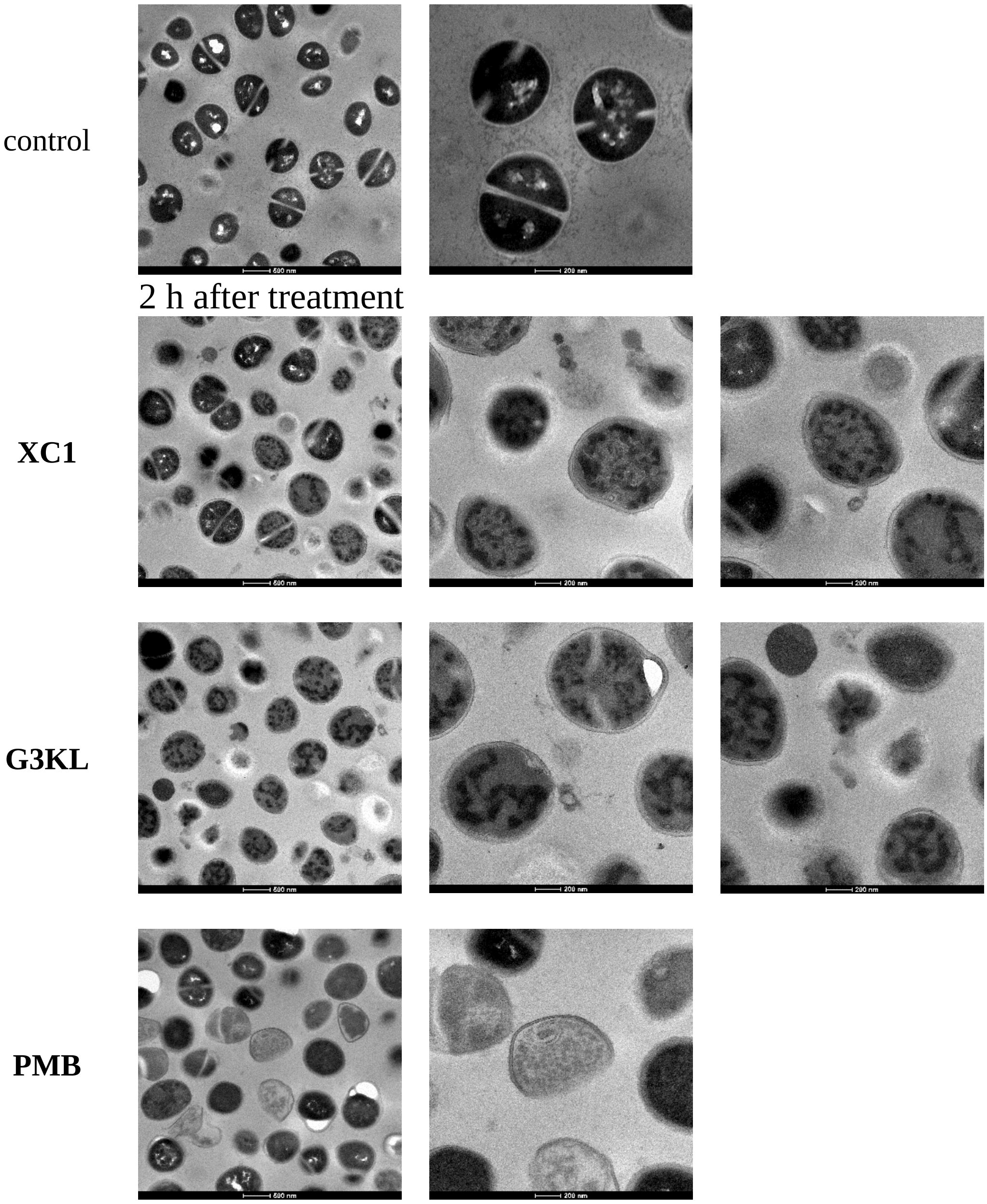

Fig. S14. TEM images of MRSA, 2 h after treatment with **XC1** (20 µg/mL), **G3KL** (20 µg/mL), and **PMB** (40 µg/mL) in MH medium at pH 8.0.

10. Quantification of bacterial binding of **G3KL-Fluo**

A single colony of *E. coli*, *A. baumannii*, *Pseudomonas aeruginosa*, *K. pneumoniae* and MRSA was grown overnight with shaking (180 rpm) in LB-broth (5 mL) at 37 °C. 100 µL of the overnight culture was regrown in 5 mL LB-broth to the exponential phase OD_{600} = 1.0 (1 x 10^9 CFU/mL). Bacteria (1 mL, OD_{600} = 1.0) were washed once with MH medium (at pH 7.4 or pH 8.0) and resuspended in 960 µL of MH medium (at pH 7.4 or pH 8.0). 100 mM NaCl, 200 mM NaCl and 300 mM NaCl was added when specified. 40 µL of 1 mg/mL **G3KL-Fluo** was then added to bacteria. After 2 hours, 180 µL of the sample were isolated and centrifuged at 12 000 rpm for 10 min. The supernatant was collected and added to a 96 well-plate (TPP, untreated, Faust Laborbedarf, AG, Schaffhausen) prior to fluorescence measurement with a Tecan instrument Infinite M1000. The plate was enabled to shake for 30 sec before measurement. The excitation wavelength used was 495 nm ± 5 nm and the emission wavelength 519 nm ± 5 nm. The assay was performed in the biosafety level 2 lab was repeated at least two times.

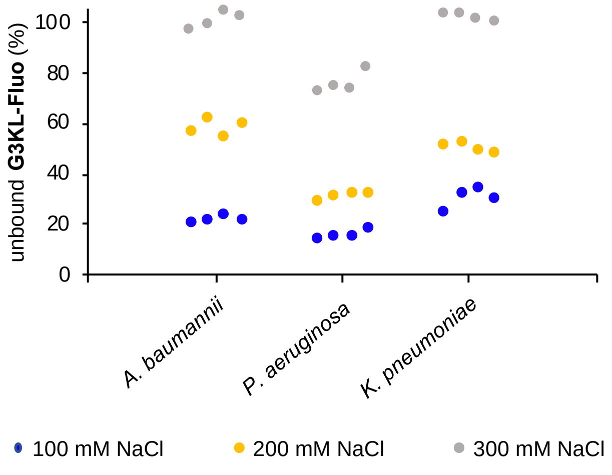

Fig. S15. Quantification of unbound **G3KL-Fluo** at pH 8.0 in the presence of 10^9 CFU/mL (OD_{600}=1) of *A. baumannii*, *P. aeruginosa* PAO1, *K. pneumoniae* and MRSA for 2 hours. Fluorescence measurement of the supernatant from treated samples at 40 µg/mL.
